# Redefining colocalization analysis with a novel phasor mixing coefficient

**DOI:** 10.1242/jcs.264388

**Published:** 2026-01-19

**Authors:** Owen F. Puls, Jesse S. Aaron, Ellen K. Quarles, Satya Khuon, Leanna R. Eisenman, Andrés Kamaid, Leonel Malacrida, Teng-Leong Chew

**Affiliations:** ^1^ Advanced Imaging Center and Integrative Imaging, Howard Hughes Medical Institute Janelia Research Campus, Ashburn, VA 20147, USA; ^2^ Advanced Bioimaging Unit, Institut Pasteur de Montevideo and Universidad de la República, Montevideo, 11400 Uruguay; ^3^ Unidad Académica de Fisiopatología, Hospital de Clínicas, Facultad de Medicina, Universidad de la República, Montevideo, 11600 Uruguay

**Keywords:** Colocalization, Phasor analysis, Multispectral imaging, Quantitative microscopy

## Abstract

The first step to probing any potential interaction between two biomolecules is to determine their spatial association. In other words, if two biomolecules localize similarly within a cell, then it is plausible they could interact. Traditionally, this is quantified through various colocalization metrics. These measures infer this association by estimating the degree to which fluorescent signals from each biomolecule overlap or correlate. However, these metrics are, at best, proxies, and they depend strongly on various experimental choices. Here, we define a new strategy that leverages multispectral imaging and phasor analysis, termed the phasor mixing coefficient (PMC). The PMC measures the precise mixing of fluorescent signals in each pixel. We demonstrate how the PMC captures complex biological subtlety by offering two distinct values, a global measure of overall color mixing and the homogeneity thereof. We additionally show that the PMC exhibits less sensitivity to signal-to-noise ratio, intensity threshold and background signal compared to canonical methods. Moreover, this method provides a means to visualize color mixing at each pixel. We show that the PMC offers users a nuanced and robust metric to quantify biological association.

## Teaser

**Highlighted Article:** By leveraging multispectral imaging and phasor analysis, this work defines a nuanced and robust metric for analyzing the association of biomolecules, the phasor mixing coefficient.

## INTRODUCTION

A perennial task across many areas of life science is to assess the extent to which two or more species of biomolecules are related. Among the various available tools, optical microscopy is particularly attractive as it can do so in a quantitative, target-specific and spatiotemporally resolved manner. However, the spatial resolution of conventional optical microscopy prevents a direct detection of molecular interactions, except in more specialized cases, such as those involving Förster resonance energy transfer (FRET) (Day, 1998; Elangovan et al., 2002; Sekar and Periasamy, 2003). More commonly, indirect approaches that quantify the similarity between separate images of each biomolecule are used, which are generally termed ‘colocalization’ methods. However, the extent to which these methods are informative is subject to many factors. Their propensity to produce misleading results is such that numerous reviews have debated their strengths, weaknesses, best implementations and overall applicability in different biological contexts (Aaron and Chew, 2018; Adler and Parmryd, 2013, 2025; Bolte and Cordelières, 2006; Dunn et al., 2011; Lopez et al., 2025; Taylor et al., 2018).

Although many methods exist, the most common are Manders' correlation coefficient (MCC) – as well as its associated channel-specific values M_1_ and M_2_ (collectively termed M_1,2_; Manders et al., 1993) – and Pearson's correlation coefficient (PCC) (Pearson, 1896). The Manders' and Pearson's coefficients quantify different aspects of color overlap in pairs of images (Aaron et al., 2018). Manders' coefficients calculate the global fraction of total signal that is found in overlapping regions. In contrast, Pearson's coefficient measures the extent to which the intensity of one image can predict the corresponding intensity of the other in the overlapping regions. Despite their differences, both coefficients infer the extent of mixing between two signals by statistical means. As a result, these measures can fail to be informative when assumptions underlying the statistics cease to hold, as elaborated below. For example, neither account for image corruption such as channel bleed-through, background signal and noise. This renders them susceptible to changes in signal-to-noise ratio (SNR), image intensity threshold selection and spatial resolution, among many other factors (Aaron et al., 2018; Costes et al., 2004; Dunn et al., 2011). Additionally, both coefficients are biased towards areas of high pixel intensity, obfuscating potentially important but subtle biological phenomena, especially when the signal overlap is heterogenous. These subtleties can be biologically informative but often cannot be accurately or sufficiently captured by these conventional colocalization coefficients. Such problems arise because both coefficients estimate the essence of colocalization – the precise mixture of biological signals – by proxy (Aaron and Chew, 2018). Instead of the broad stroke, cumulative measures that underpin Pearson's and Manders' coefficients, a more informative quantification of signal mixing should capture the precise contribution of each fluorescent signal at each pixel. To accomplish this, we turned to multispectral imaging, a technique powerfully tuned to characterizing the mixing of fluorescent signals.

Multispectral imaging is a common feature available in commercial confocal microscopes (Zimmermann, 2005; Garini et al., 2006). Rather than acquiring a single image of each biomolecule using predefined excitation and emission filters, multispectral imaging acquires a lambda stack containing fluorescence spectra at each pixel position in the image. This relatively simple technique can discriminate fluorophores of similar spectra through various spectral ‘unmixing’ approaches (Dickinson et al., 2001). Moreover, this information can be used to precisely remove spurious factors, such as bleed-through and autofluorescence, that can produce misleading results (Fereidouni et al., 2012; Vallmitjana et al., 2022). This allows subsequent analysis to operate purely on the signals of interest.

One approach for unmixing and analyzing multispectral images is the phasor method (Jameson et al., 1984; Weber, 1981). In contrast to other techniques such as linear regression, the phasor approach is distinctly advantageous due to its computational simplicity, with little or no *a priori* information needed (Fereidouni et al., 2012). This method is utilized extensively in the context of fluorescence lifetime imaging (FLIM) (Digman et al., 2008; Ranjit et al., 2018b; Stringari et al., 2011). It has more recently been demonstrated to be equally powerful for spectral unmixing (Fereidouni et al., 2012; García et al., 2023; Hedde et al., 2021; Vallmitjana et al., 2021; Yao et al., 2022). We therefore reasoned that the phasor representation itself contains vital information that describes the richness and complexity of fluorescence signal mixing.

In this paper, we report on the design of a novel phasor mixing coefficient (PMC) that leverages spectrally resolved microscopy data to address the disadvantages of traditional methods. By combining multispectral imaging and phasor analysis, we calculate the contributions of individual signals explicitly at each pixel in the image. This allows the user to understand the often-subtle variation in the mixing of biological signals. We further evaluate the performance of the PMC and demonstrate why it can be a more biologically informative coefficient. In the following sections, we outline the mathematics behind this method and highlight its efficacy in various biological contexts.

## RESULTS

### Motivation

**Fig. 1.**
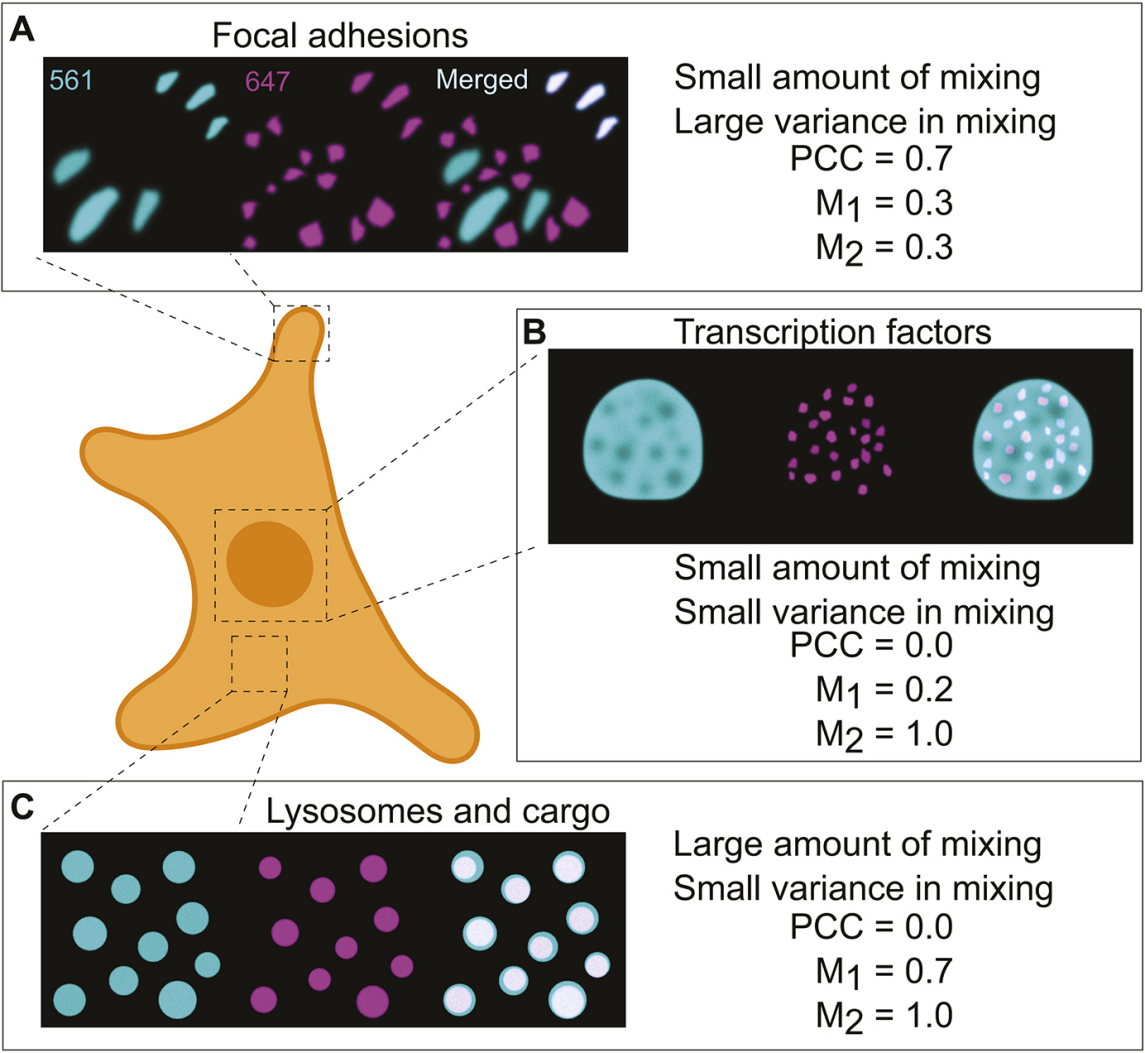
**Simulated single-color images of three different biological contexts demonstrate how the association of biomolecules can exhibit a wide range of degree and homogeneity.** (A) A protein (magenta) localizes to nascent focal adhesions but isolates from mature adhesions (cyan), leading to significant association in some areas of the sample and minimal association elsewhere. PCC misses the areas of minimal mixing as it is only defined over the intersection. (B) Transcription factors (magenta) completely associate with the nucleus (cyan), although parts of the nucleus contain no transcription factors. PCC misrepresents this situation by indicating no correlation. M_1,2_ correctly identifies the disparity in the association. (C) Lysosomes (cyan) traffic different amounts of cargo (magenta). The association is largely uniform. PCC misstates the association. In this case, the relatively small difference in M_1,2_ is difficult to interpret. Here, 561 and 647 refer to the peak excitation wavelengths (in nm) of simulated fluorophores used to label these structures.

Before defining and testing this new method, it is helpful to demonstrate the potential shortcomings of the PCC and/or M_1,2_ with regards to capturing biological subtlety. Using simulated images that reflect common biological scenarios, Fig. 1 illustrates some of these limitations. To start, molecular associations do not always occur homogenously within a living system. For example, in Fig. 1A, consider a hypothetical protein that preferentially associates with nascent but not mature focal adhesions. This translates into both low overall color overlap and large variation in the degree of overlap across the cell. In this case, M_1,2_ report minimal signal overlap. This is because M_1,2_ report the average amount of overlap between the two proteins. They do not identify the heterogenous extent of overlap between the nascent and mature focal adhesions. Conversely, the PCC is reasonably high because it only considers the nascent adhesions. A more complete biological picture may be found by considering both pieces of information together. Yet, most users do not often make use of this nuance when reporting colocalization values. To make matters worse, the next two examples illustrate contexts in which even the combination of these metrics falls short.

Another common scenario is when one molecule consistently co-occurs with another, but not reciprocally, such as transcription factors within a nucleus (Fig. 1B). The degree of overlap is consistent but confined to a small portion of the total nuclear area. In this case, M_1,2_ provide an intuitive description of the overlap in both channels. In contrast, the low PCC value contradicts the self-evident association of the transcription factors with the nucleus. Additionally, two signals can co-occur with each other, but to a varying degree throughout the cell. A good example of this phenomenon is the heterogenous overlap between lysosomes and their cargo (Fig. 1C). In this case, the small difference between M_1,2_ values is difficult to interpret biologically. Compounding this confusion, the PCC further indicates essentially no relationship between the lysosomes and their cargo. In these two cases, we see common scenarios where the PCC and M_1,2_ coefficients appear to contradict each other, leaving biologists to choose which limited measure best captures the biological reality.

The shortcomings presented here exemplify the perils of trusting these coefficients and disregarding the biological circumstances at hand. They argue that, at minimum, a more encompassing description is warranted. It is with this in mind that we defined the PMC.

### The phasor method

**Fig. 2.**
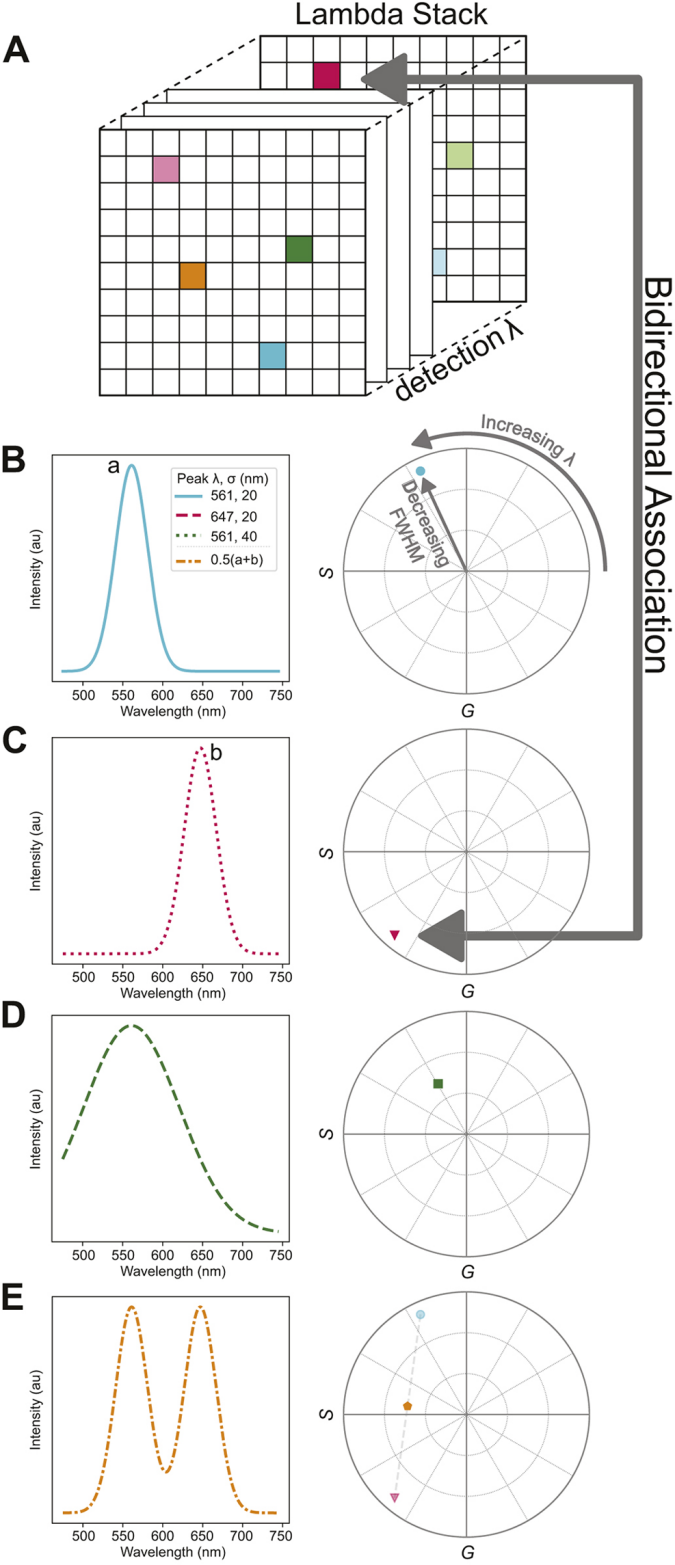
**Phasor analysis transforms a spectrum into a point in 2D space for each pixel in a multispectral image.** (A) Multispectral microscopy outputs an image stack (‘lambda stack’) in which each individual frame corresponds to a detection wavelength. The information encoded at each *x*-*y* location (pixel) reflects the detected spectrum. (B–D) The phasor method maps each spectrum (left) to a point in phasor space (right). The angular position of this point reflects the peak wavelength: increasing lambda moves the point counterclockwise (compare B and C). The radial position reflects the full-width half-max (FWHM): increasing the FWHM moves the point towards the origin (compare B and D). This mapping is bidirectional, meaning each phasor point corresponds to exactly one spectrum at one pixel. (E) If a pixel contains a mixture of two spectra, then it is mapped to a phasor point that sits on the line connecting those spectra. au, arbitrary units.

In conventional fluorescence images, every pixel contains a single intensity value. In contrast, multispectral images contain intensities across a range of wavelengths at each pixel (Fig. 2A). Visualizing this three-dimensional dataset can be challenging. To ameliorate this issue, a phasor plot can be used. The phasor representation captures a fluorescence spectrum at each pixel by describing its peak wavelength and spectral width (Fereidouni et al., 2012; Malacrida et al., 2021). It does so by calculating two specific numbers (*G* and *S*) from the spectrum (see Eqn 3 in the Materials and Methods) that can be plotted as a phasor point inside a circle on a two-dimensional (2D) graph, as shown in Fig. 2B–E.

Firstly, in this approach, spectra with longer peak wavelengths are positioned at larger angles in the phasor plot, whereas spectra with shorter peak wavelengths are represented at shorter angles. (Fig. 2B,C). For example, compare the solid blue spectrum (Fig. 2B; peak wavelength of 561 nm), the dotted magenta spectrum (Fig. 2C; peak wavelength of 647 nm) and their corresponding phasor points (blue circle and magenta triangle, respectively). Secondly, narrower spectra are located further from the origin of the phasor plot, with a maximum radius of one, whereas wider spectra are located closer to the center: compare the solid blue and dashed green spectra and their corresponding phasor points (blue circle and green square, respectively; Fig. 2B,D). These features of the phasor plot, in essence, describe the essential characteristics of any spectrum. These same features, by extension, are equally useful to describe the mixing of two spectra: any pixel that contains a combination of two colors will fall on a line connecting the phasor points of each component (Fig. 2E). Moreover, the position along this line explicitly defines the relative proportion of the two component colors. In short, phasor analysis constitutes a foundation for more information-rich colocalization analysis.

**Fig. 3.**
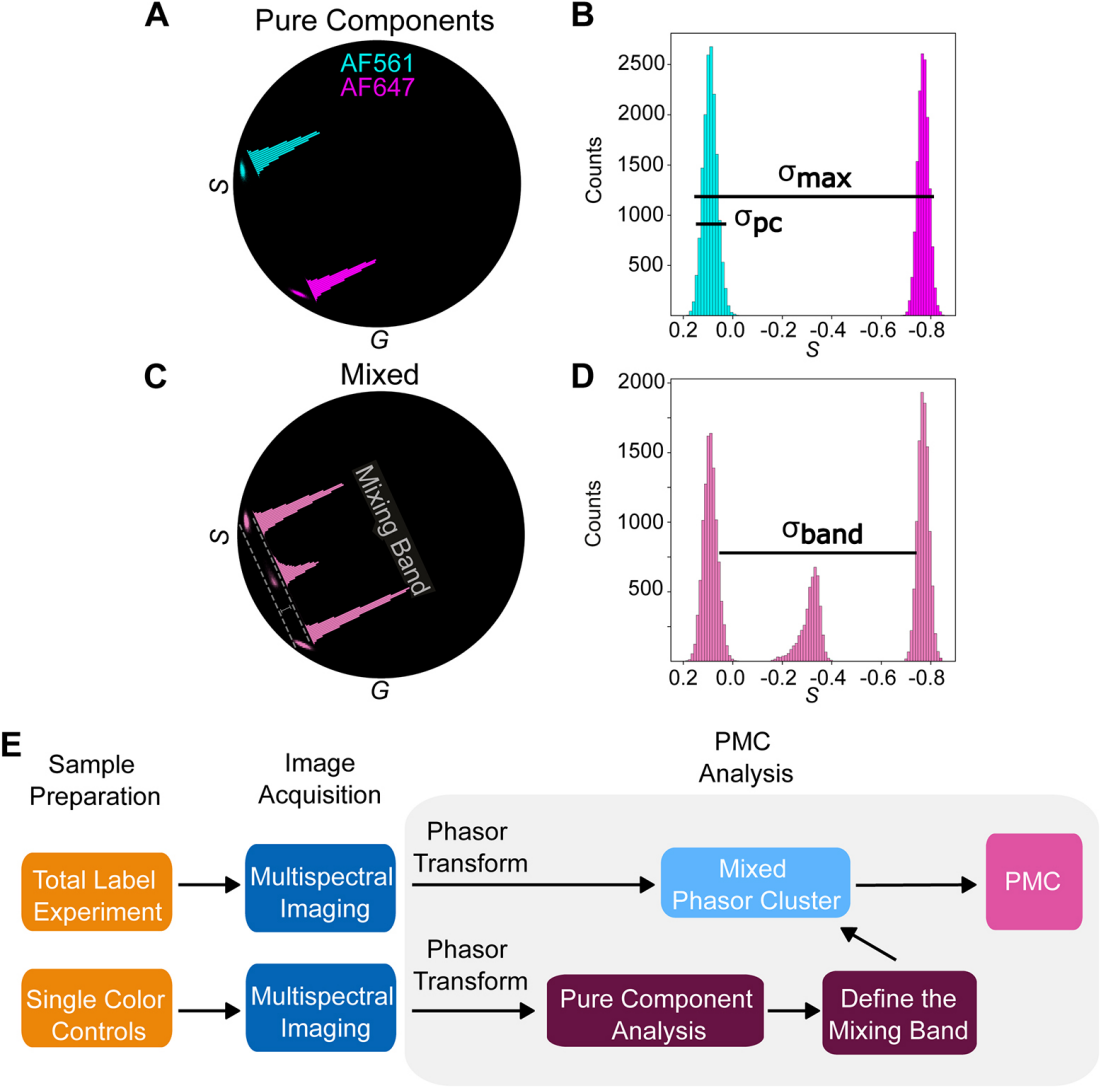
**Clusters of phasor points can reflect populations of single fluorophores or mixtures of multiple fluorophores.** (A) Phasor clusters from two separate simulated images labeled with either AF561 (cyan) or AF647 (magenta). The size of the cluster reflects the SNR of the image. These are referred to as ‘pure components’ (PCs). The correspondingly colored histogram overlays represent the density of phasor points projected along the *S* (vertical) axis. (B) The overlaid histograms from A shown in more detail. The size of the individual clusters (*σ*_pc_) and that of the pseudo cluster formed by concatenating the individual pure component clusters (*σ*_max_) are determined as described in the Materials and Methods. (C) The phasor plot from a simulated image containing structures labeled with both AF561 and AF647. Note the overlaid histogram, similar to that in B, showing the density of phasor points. Some pixels contain only one label, and thus sit near the center of the pure component clusters; other pixels contain a mixture of the two labels, and thus sit on the line connecting the pure components. This line is referred to as the ‘mixing band’. The bounds of the mixing band are marked by dashed lines, and the bracket indicates the measured width of the mixing band, as described in the Materials and Methods. (D) The histogram of the *S* coordinates of the phasor points within the mixing band in C are shown in more detail. *σ*_band_ reflects the spread of all phasor points between the centers of the pure components. Together, *σ*_pc,_ *σ*_max_ and *σ*_band_ are used to calculate PMC_2_ as described in the Materials and Methods. (E) Flowchart outlining the workflow for multispectral mixing analysis using the PMC. Single-label controls and samples containing both labels are prepared as required. These samples are all imaged under the same settings using multispectral imaging. Finally, PMC analysis (gray box) processes the resulting lambda stacks to produce the PMC as described in the text. The steps within the gray box require no user input outside calling the relevant functions as illustrated in the analysis code.

A phasor plot can be made by plotting (*G*,*S*) coordinates for every pixel in an image. Pixels with similar spectra will form a cluster in the phasor plot (Fig. 3A) regardless of their physical location in the image. As a result, the brightness of regions within the phasor plot reflects the number of pixels at that location (Fig. 3A,C). If each pixel in an image contains only one of two distinct colors (i.e. zero colocalization) then the phasor plot will consist of two spatially segregated ‘pure component’ (PC) clusters of points (Fig. 3A,B). If, however, the image contains pixels with mixtures of two colors (i.e. colocalization), the phasor plot will also contain points that fall along the line connecting the two centers (*G_pc_*, *S_pc_*) of these clusters (Fig. 3C,D; see Materials and Methods). The locations along this line provide a means to precisely quantify the color mixing, as described below (Ranjit et al., 2019; Torrado et al., 2022; Vallmitjana et al., 2020).

A pixel containing fractions, *f*_1_ and *f*_2_, of two pure components (*pc*1 and *pc*2) can be found at the phasor point (*G_mix_, S_mix_*) (Eqn 1):
(1)

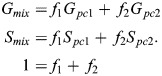


Conservation of signal imposes the constraint 1=*f*_1_+*f*_2_. This mathematical convenience affords a simple way to calculate the proportion of two colors at each point in an image (Torrado et al., 2022). This pixel-level measurement of color mixing thus constitutes the underlying principle of our phasor plot-driven colocalization coefficient, PMC.

Phasors also exhibit reciprocity, meaning each (*G*, *S*) point uniquely corresponds to a single pixel in the multispectral image (as indicated by the bidirectional arrow in Fig. 2A) (Digman et al., 2008; Malacrida et al., 2021). This means that any analysis performed in phasor coordinates can be mapped back to the original image (Torrado et al., 2022). We leveraged this feature for powerful visualization capabilities that highlight biological aspects that could otherwise be overlooked.

### Measuring colocalization in phasor space

To accurately quantify the relationship between two biomolecules in an image, it is imperative to separate pixels of interest from the background. The phasor plot offers a uniquely effective and simple method to do so independently of intensity threshold application, as is generally used in other methods. Briefly, we create a ‘mixing band’ around the line connecting the pure components to eliminate background pixels from consideration. The width of this band is defined by the diameter of the pure component clusters, which is related to the SNR of the image (Cutrale et al., 2017; Ranjit et al., 2018a). Although several parameters are required to define the mixing band, we found that a single value for each was sufficient to successfully analyze all data in this study. Full details can be found in the Materials and Methods. By only considering pixels that fall within the mixing band – that is, pixels with sufficient SNR – the phasor plot provides a fully data-informed way to focus on pixels of interest (dashed lines in Fig. 3C). This analysis imparts an important advantage over Manders' and Pearson's analyses, which typically rely on a prior user-defined threshold method that can affect their values and is prone to bias (Aaron et al., 2018; Dunn et al., 2011).

An unbiased ability to extract biologically relevant pixels sets the framework for accurately measuring color mixing. Recall that the position of each phasor point along the axis of the mixing band fully describes the relative amount of each fluorophore in the corresponding pixel: the fractions *f*_1_ and *f*_2_ (Eqn 1). We then define a new quantity *f_mix_* at each pixel (Eqn 2):
(2)

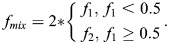


The maximum value *f_mix_*=1.0 indicates balanced 50/50 mixing of the fluorophores, whereas its minimum *f_mix_*=0.0 indicates that the pixel contains only one pure component. We then survey all *f_mix_* values, which we term the ‘mixing distribution’ (see Materials and Methods and Fig. S2). The mean (*µ*) of the mixing distribution forms the first element of the PMC. This value measures the average mixing of the two fluorophores across the image. In biological terms, a mean of 1 indicates that the two target molecules are in equal proportion to each other throughout the image, whereas a value of 0 indicates complete spatial exclusion.

This average mixing measurement is valuable. However, when considered by itself, it suffers from the same image-wide generalization that renders PCC and M_1,2_ potentially uninformative (Fig. 1). We therefore constructed a second element of the PMC to describe the mixing consistency throughout the image. In practice, biological interactions tend to be heterogeneous across regions. This information gap can be filled by further analysis of the phasor plot. While the standard deviation of *f_mix_* could be used, its value is highly sensitive to SNR, thus obfuscating its biological interpretation. We therefore define a second feature (*σ*) that quantifies the heterogeneity of color mixing in a more informative manner. It utilizes a data-driven normalization whereby *σ* takes a value of 1 when the mixing is fully homogenous subject to the SNR of the image. Conversely, a value of 0 implies maximal heterogeneity that coincides with complete spatial exclusion. A full mathematical definition is given in the Materials and Methods. Taken together, we define [*μ*, *σ*]=[PMC_1_, PMC_2_]≡PMC. PMC_1_ and PMC_2_ are also referred to here collectively as PMC_1,2_. The complete workflow – from imaging to analysis – is graphically summarized in the flowchart in Fig. 3E.

### The PMC is more biologically informative than PCC and M_1,2_

**Fig. 4.**
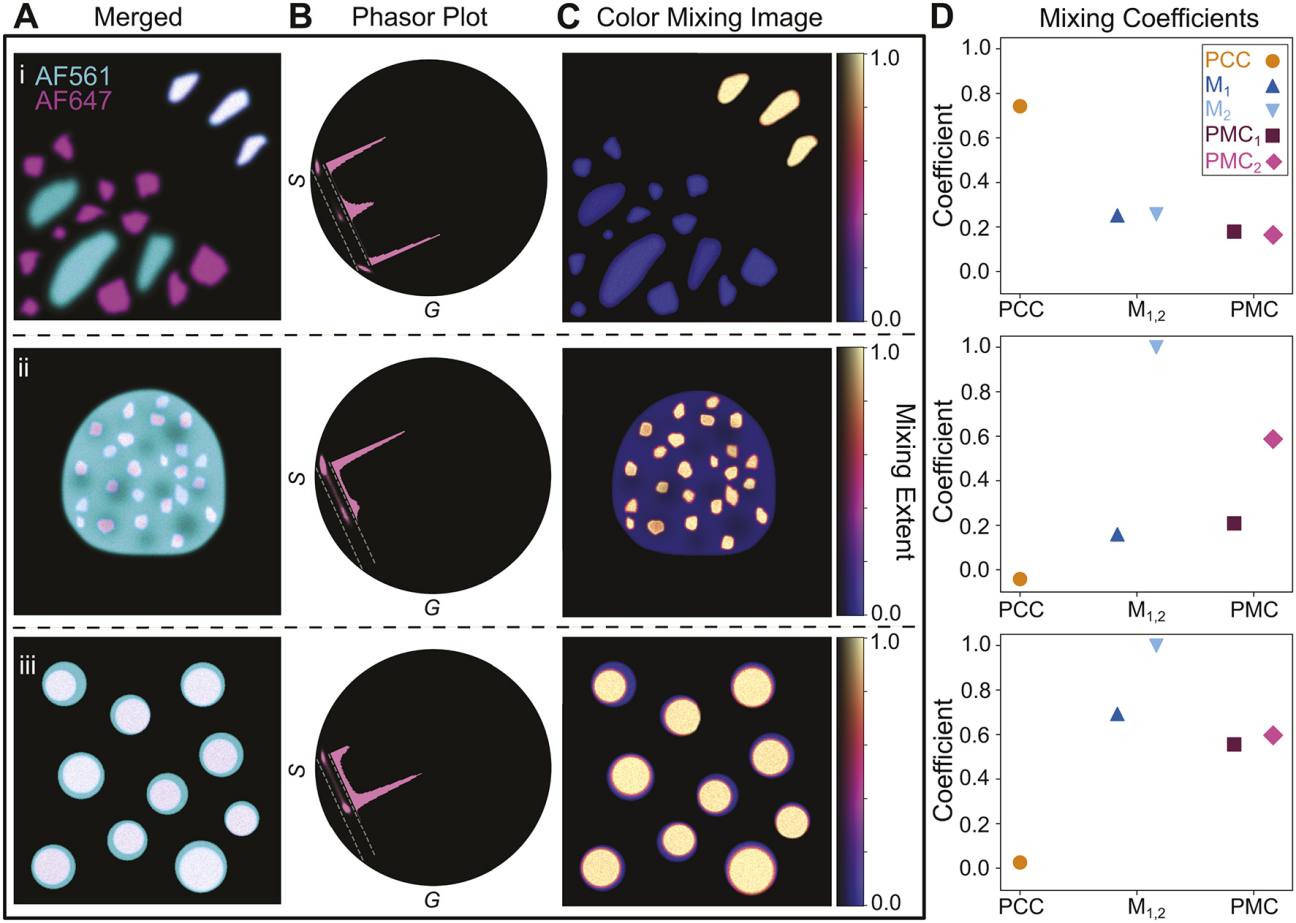
**The PMC quantifies the mixing extent and homogeneity of fluorescent signals in different biological contexts.** (A–D) Analysis of the simulated examples, where each row (i–iii) corresponds to an example in Fig. 1A–C. (A) Merged pseudo-color images from the simulated examples. Multispectral images were simulated from intensity image inputs, and subsequently, single channels were extracted as described in the Materials and Methods. (B) Phasor plots for each example. The dashed gray lines indicate the bounds of the mixing band. The overlaid histograms display the *S* coordinate of the underling phasor cluster defined within the mixing band. (C) Color mixing images for each example. The colormap represents a 2D look-up table (LUT). The color reflects the extent of mixing: hotter colors indicate more mixing. The brightness corresponds to the total amount of signal. More details can be found in the Materials and Methods. (D) PCC, Manders' coefficients (M_1_ and M_2_) and the PMC (PMC_1_ and PMC_2_) for each example.

To see how this new coefficient remedies the pitfalls of Pearson's and Manders' coefficients, we first return to the examples presented in Fig. 1. In Fig. 4, we show the pseudo-color merged images from each example (Fig. 4Ai–iii), along with phasor plots from simulated spectra (Fig. 4Bi–iii) (see Materials and Methods). The white dotted lines in Fig. 4B indicate the mixing bands, as described above. The PMC values for each example are plotted along with the corresponding PCC and M_1,2_ values (Fig. 4D).

#### Interpreting PMC values

In each example, the phasor plots and PMC quantities more comprehensively described what was evident from the underlying structure (Fig. 4). For the scenario shown in Fig. 4Ai (scenario i), a small PMC_1_ value (0.19) indicated that only a small proportion of the two proteins associate within the cell; meanwhile, the small PMC_2_ value (0.16) reflected the bimodal nature of this association. Indeed, this dichotomous relationship cannot be gleaned from the PCC or M_1,2_ values (see Fig. 1). For the scenario shown in Fig. 4Aii (scenario ii), the polar difference between the small PMC_1_ and the larger PMC_2_ values (0.20 and 0.58, respectively) captured the homogenous association between the two molecules in confined regions. In other words, although the transcription factors only occupy a small proportion of the nucleus, the association is consistent. Comparing these two scenarios revealed the increased homogeneity in the latter that is captured by PMC_2_. For the scenario shown in Fig. 4Aiii (scenario iii), the pervasive and relatively homogeneous mixing between lysosomes and cargo was identified. In this case, both PMC_1_ (0.57) and PMC_2_ (0.60) are reasonably large, indicative of the fact that each lysosome carries some amount of cargo.

Comparing the PMC values across these examples highlights how the metric captures diverse states of mixing. The PMC_1_ values are similar in scenarios i and ii because the relative number of pixels that primarily contain only one of two labels – either cyan or magenta – is similar in the two cases. However, PMC_2_ captures the difference between these examples. In scenario i, there are pixels containing both signals, as well as pixels that contain only cyan or magenta. In scenario ii, there are no pixels that contain only magenta. This results in a higher PMC_2_ value in scenario ii than in scenario i precisely because the homogeneity is higher. Alternatively, the PMC_2_ values are similar in scenarios ii and iii because both examples exhibit similar kinds of mixing: regions with only one (cyan) signal and regions with high levels of mixing (cyan and magenta). However, the relative contributions of these regions are different between the examples. In scenario ii, the unmixed region is larger than the mixed region; the opposite is true in scenario iii. As a result, the overall extent of mixing is different between the two examples, which is captured by differences in PMC_1_ values. Unlike PCC and M_1,2_, the PMC provides two complementary sets of information that more thoroughly describe the biological reality.

To further understand how the PMC quantifies mixing, it is helpful to consider a perturbation to the example in Fig. 1A. If a small number of magenta objects not associated with focal adhesions were removed, PMC_1_ would remain largely constant because the total amount of mixing has not markedly changed. However, removing these objects would significantly increase the PMC_2_ value. This is because the proportion of unmixed colors is reduced, making the overall system more homogeneous. Such a perturbation highlights how sensitively the interplay between the PMC values reflects biological variation, thus illustrating their unique experimental utility across a range of studies.

#### Color mixing image

In addition to its multifaceted quantification, the phasor method also offers a powerful way to visualize mixing. Recall that *f*_*mix*_ is defined at every pixel. As a result, we can map this value back to the image in a way that preserves the original total intensity information, combined with the calculated mixing (see Materials and Methods). This color mixing image (CMI; Fig. 4Ci–iii) is a unique data exploration tool that highlights where and to what degree mixing occurs. It constitutes an information-rich dataset that can support not only visualization but also subsequent image processing, analysis and interpretation. More importantly, it eliminates the ambiguity of pseudo-color merged images that are subject to errors in human perception (Taylor et al., 2017). For instance, the CMI unambiguously highlighted the nascent focal adhesions (Fig. 4Ci). In this case, the mixing at the nascent adhesions is largely uniform. However, in instances where there is more gradation in mixing, this tool can provide a means to isolate specific subpopulations of biomolecular associations along this gradation. For example, note the varying mixing extent across transcription factors in Fig. 4Cii. This reflects the distribution of points along the mixing band, ranging from minimal to complete mixing. This informative feature is unavailable in other methods.

### Applying the PMC to biological data

**Fig. 5.**
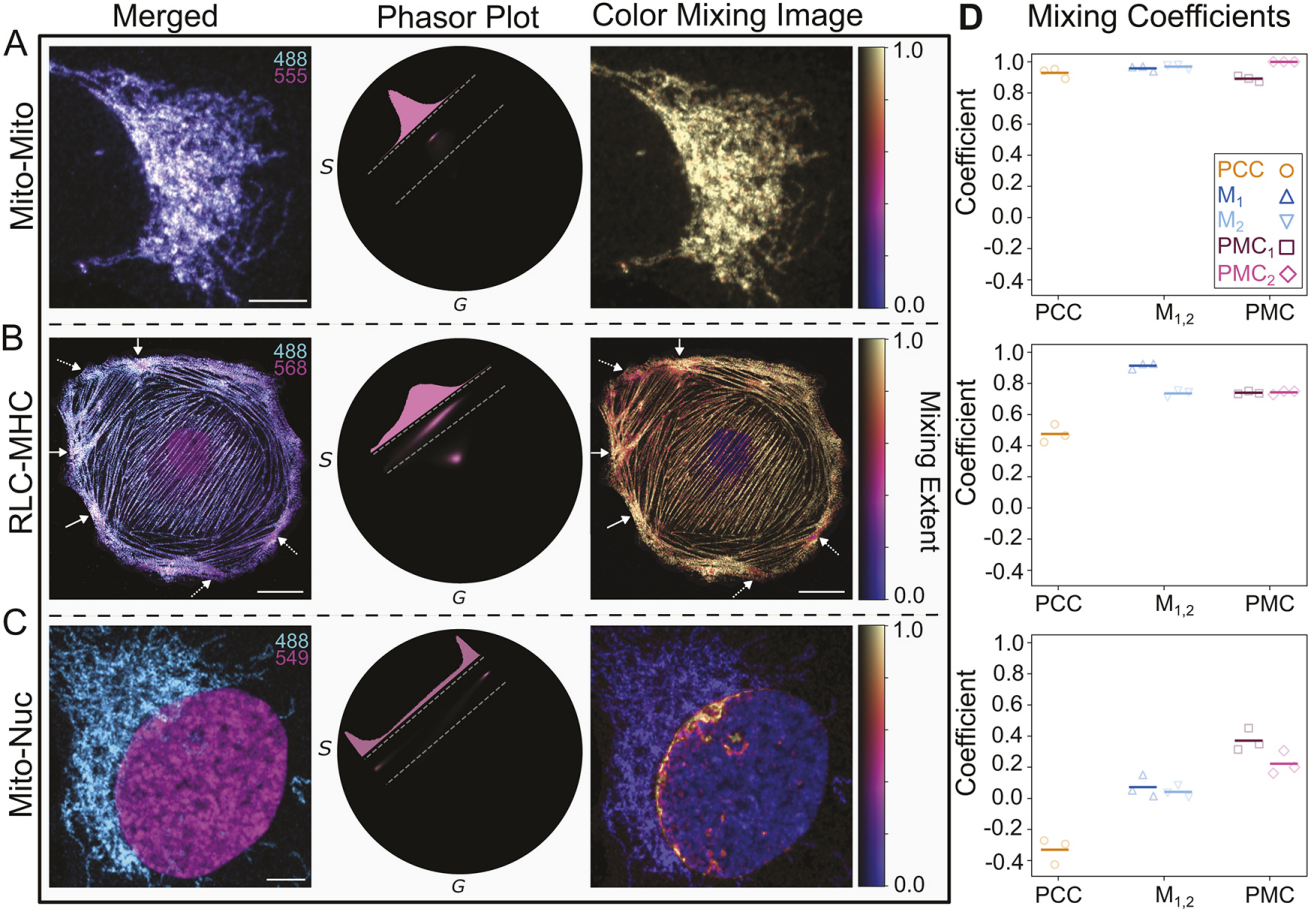
**The PMC recapitulates the expected quantification across a wide range of biomolecular association.** (A–C) Examples illustrating how the PMC quantifies known high (A), moderate (B) and low (C) biomolecular associations. (A) Color mixing analysis of U2OS cells with mitochondria double-labeled by using an anti-TOMM20 antibody with both AF488-conjugated and AF555-conjugated secondary antibodies. The merged pseudo-color image (left; AF488 in cyan; AF555 in magenta) was extracted from the multispectral image. Scale bar: 10 µm. The phasor plot (middle) exhibits a single narrow cluster near the midpoint of the mixing band. The CMI (right) highlights essentially all mitochondria. The brighter regions near the nucleus reflect a slight difference in depth throughout the cell. (B) Color mixing analysis of a PtK2 cell with MHC labeled with AF488 and phosphorylated RLC labeled with AF568. The merged pseudo-color image (left; AF488 in cyan, AF568 in magenta) was extracted from the multispectral image. Scale bars: 20 µm. The phasor plot (middle) exhibits a single broad cluster centered near the midpoint of the mixing band. The cluster near the origin corresponds to the significant number of background pixels that are excluded from the mixing band. The CMI (right) highlights regions with significant mixing (solid white arrows) and minimal mixing (dashed white arrows). (C) Color mixing analysis of U2OS cells with mitochondria labeled with anti-TOMM20 antibody and an AF488-conjugated secondary antibody, and with nuclei labeled with Hoechst–JF549. The merged pseudo-color image (left; AF488 in cyan, JF549 in magenta) was extracted from the multispectral image. Scale bar: 10 µm. The phasor plot (middle) exhibits a largely bimodal cluster in which the two lobes sit near the two PCs. The mixing image (right) is largely uniformly cold, with only a region near the edge of the nucleus displaying any mixing. This small region reflects areas where the mitochondria extend over (or under) the nucleus but are still captured in the optical section. In the phasor plots, dashed gray lines indicate the bounds of the mixing band. The overlaid histograms display the *S* coordinate of the underling phasor cluster defined within the mixing band. (D) PCC, Manders' coefficients (M_1_ and M_2_) and the PMC (PMC_1_ and PMC_2_) for each example in A–C (from top to bottom). PCC and M_1,2_ were calculated after applying an intensity threshold determined by the Otsu method (see Fig. S4). Importantly, PMC_1,2_ capture the known association moving from high (top, A) to low (bottom, C) mixing. Individual *N*=3 points correspond to individual samples; line marker indicates the mean value.

To assess the performance of the PMC in actual biological data, we first examined U2OS cells wherein mitochondria had been doubly labeled with Alexa Fluor (AF) 488 and AF555 (Fig. 5A). This sample is expected to exhibit nearly complete and homogeneous color mixing. In this simple scenario, PMC_1,2_=[0.89, 1.0] reflected this accordingly (Fig. 5D, top). We then tested the PMC in a more complex situation. In this case, non-muscle myosin II heavy chain (MHC) in PtK2 cells was labeled with AF488, while its phosphorylated regulatory light chain (RLC) was labeled with AF568 (Fig. 5B). This sample contained subtle variation in color mixing, where RLC could be phosphorylated to varying degrees in different regions in the cell (white arrows in Fig. 5B). As a result, PMC_1,2_=[0.74, 0.74] reflected this biological subtlety by displaying less overall mixing and more heterogeneity (Fig. 5D, middle). Finally, we probed how the PMC would function in the opposite extreme case of near-complete spatial exclusion of two molecules. Nuclei and mitochondria in U2OS cells were labeled with Hoechst–JF549 and AF488, respectively (Fig. 5C). As expected, PMC_1,2_=[0.38, 0.21] indicated significantly reduced color mixing and accompanying high heterogeneity (Fig. 5D, bottom). This interpretation coincided with what one would expect from the measurements using PCC and M_1,2_ (Fig. 5D; Fig. S4).

However, closer examination of the phasor plots revealed the additional information provided by the PMC (Fig. 5A–C, middle column). When comparing the double-labeled mitochondria image with the RLC and MHC labeling image, both examples exhibited relatively high mixing. This is evidenced by the high density of phasor points between the pure components. They therefore registered high PMC_1_ values. However, the double-labeled mitochondria sample naturally showed more homogeneous mixing than did the two different subunits of myosin. As a result, the dual mitochondria phasor points were more tightly clustered than those in the myosin example (Fig. 5A,B, middle). The divergent PMC_2_ values, therefore, reflected this important biological difference. Conversely, the mitochondria–nucleus sample exhibited a low level of mixing, as expected. Nearly all phasor points were segregated near the two pure components, thus diminishing both PMC_1_ and PMC_2_ values. See the Materials and Methods and Fig. S2 for further details.

As mentioned above, phasor analysis contains pixel-specific information that can be used to generate a CMI. This heatmap elucidates more biological details by identifying regions that correspond to a specific degree of mixing. In the examples above, the double-labeled mitochondria image exhibited high mixing levels throughout the image (Fig. 5A, right). In the case of mitochondria–nucleus CMI (Fig. 5C, right), few regions indicated any appreciable mixing. On the other hand, the RLC and MHC example highlights a more subtle behavior. The CMI (Fig. 5B, right) reveals regional myosin phosphorylation ranging from maximal (solid white arrows) to nearly zero (dashed white arrows) (Fig. 5B, left and right). This allows end users to set biological thresholds to differentiate regions with a specific range of molecular associations. Moreover, the CMI allows users to describe nuanced biological behaviours through an informed choice of colormap. Fig. S3 illustrates how this choice can highlight (1) areas of high mixing (2) areas of spatial exclusion and (3) the gradation between these states, all from the same image. Taken together, these three examples demonstrate how this method distinguishes biological subtlety.

### The PMC exhibits less susceptibility to thresholding methods than PCC or M_1,2_

**Fig. 6.**
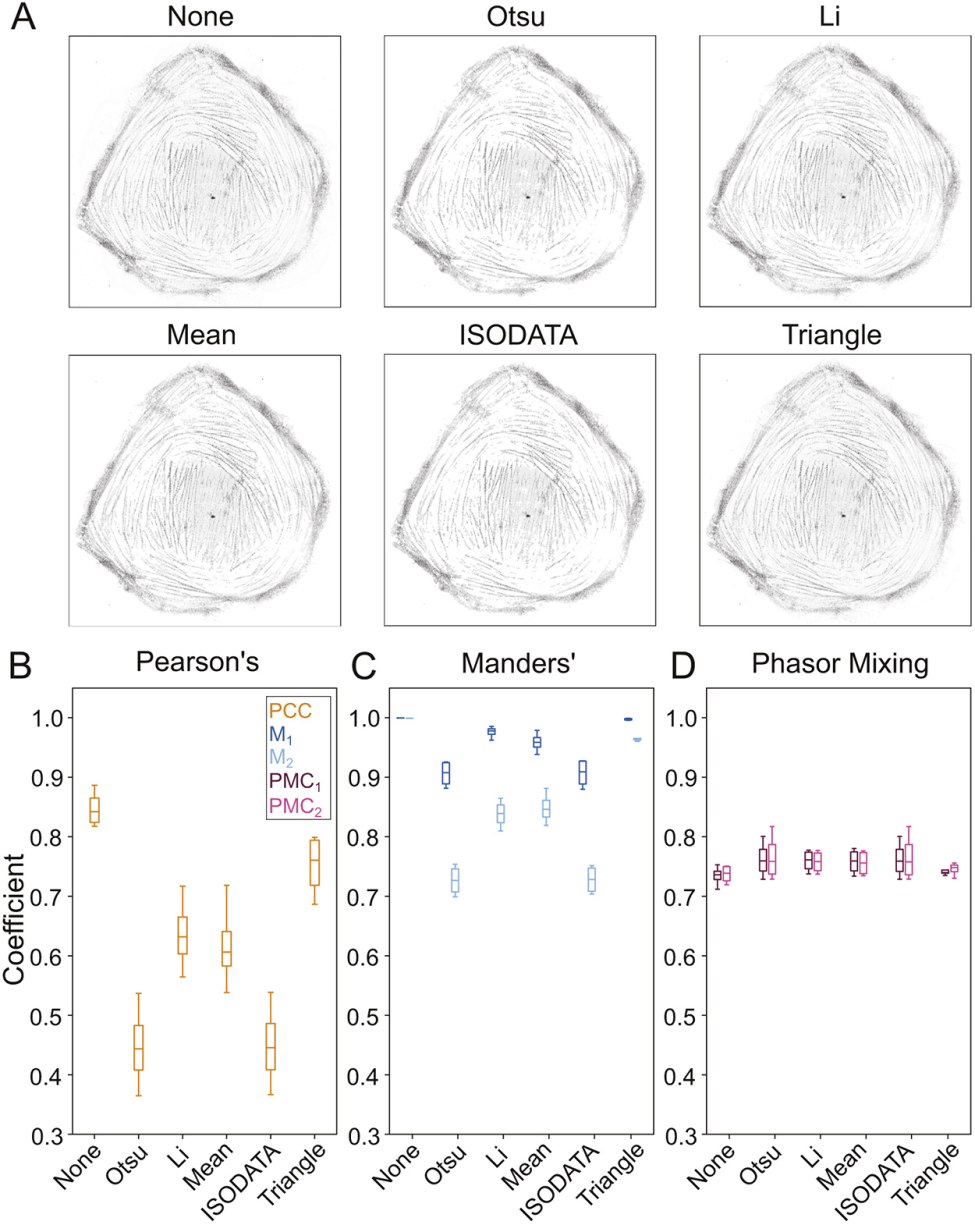
**The PMC exhibits less sensitivity to thresholding method than Pearson's or Manders’ coefficients.** (A) Representative summed intensity images of the image data from Fig. 5B after applying an intensity threshold as determined by one of the indicated methods (or no threshold). (B) PCC measurements for the various intensity thresholding methods illustrated in A. PCC varies significantly depending on the choice of method. (C) M_1,2_ measurements for the various intensity thresholding methods illustrated in A. M_1,2_ is not a representative measure without intensity thresholding. Although it varies less than PCC, it still varies by 20% across the choice of method. (D) PMC measurements across the thresholding methods illustrated in A. Both PMC_1_ and PMC_2_ exhibit substantially less sensitivity to the choice of method. Data in B–D are from *N*=4 samples and are presented as boxplots showing the interquartile range (box), median (line) and the most extreme data point(s) within 1.5x the Inner-quartile range (IQR) above the third and below the first quartile (whiskers). Key in B also applies to C and D.

As discussed in the Introduction, both Pearson's and Manders' coefficients are perilously sensitive to the choice of thresholding method (Aaron et al., 2018; Dunn et al., 2011). This can introduce significant bias into any colocalization measurement, as pixels may be included (or excluded) depending on the user's discretion. To examine whether the PMC avoids this sensitivity, we applied various thresholding methods (Li and Lee, 1993; Otsu, 1979; Ridler and Calvard, 1978; Zack et al., 1977) and calculated the coefficients using the data from Fig. 5B (see Fig. 6A for representative intensity images after applying the threshold from each method). Fig. 6B,C illustrates that, unlike PCC (Fig. 6B) and M_1,2_ (Fig. 6C), PMC_1,2_ (Fig. 6D) exhibits minimal sensitivity to threshold choice. In fact, the PMC functions consistently even without intensity thresholding. This underscores the important ability of the PMC to address one of the core weaknesses of traditional colocalization metrics.

Although phasor analysis is intensity-agnostic, threshold application can still remove spurious signals in regions that are not under consideration. For example, in the images from Fig. 5A, the nucleus exhibited some non-specific staining by both probes, although the total intensity was sufficiently low to be almost imperceptible (labeled in Fig. S5). The phasor coordinates for these pixels occupy a similar space to those of the foreground pixels in the mitochondria (Fig. S5). An intensity-based threshold will eliminate the effects of these spurious signals in the PMC calculation. In this example, the overall effect on the PMC is minimal (<5%; Fig. S5). Our method, therefore, does not preclude the use of existing tools to mitigate the inherent imperfections of biological experiments, so long as they are used appropriately.

### The PMC exhibits less susceptibility to SNR than PCC or M_1,2_

**Fig. 7.**
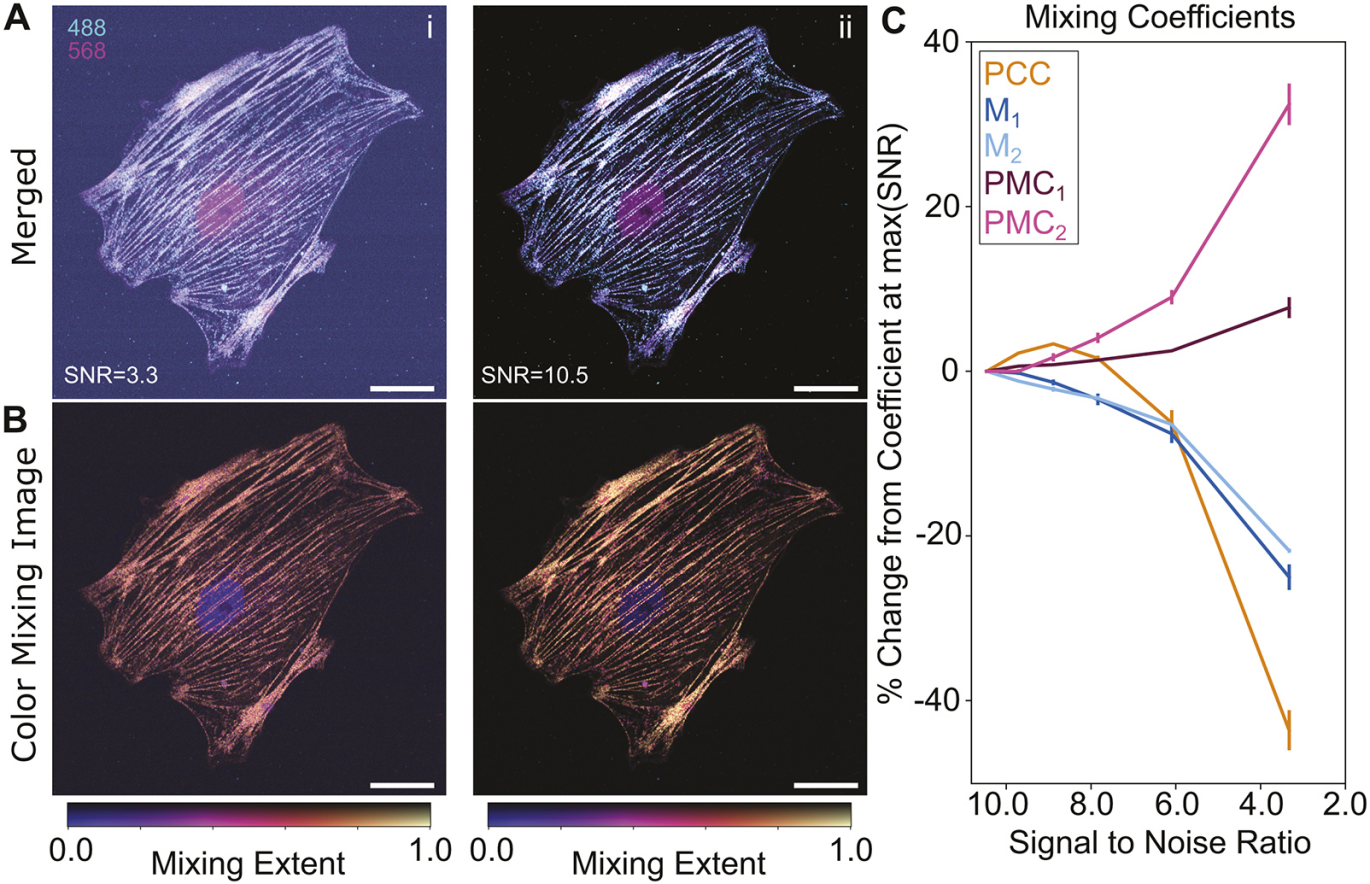
**The PMC exhibits less sensitivity to SNR than Pearson's or Manders’ coefficients.** (A) Merged pseudo-color images (extracted from multispectral images) of PtK2 cells with MHC labeled with AF488 (cyan) and RLC labeled with AF568 (magenta) at low (i) and high (ii) SNR. Scale bars: 20 µm. (B) The mixing images corresponding to the low (left) and high (right) SNR images in A. Despite the significant increase in noise, the low-SNR mixing image largely highlights the same regions as the high-SNR image. Scale bars: 20 µm. (C) Quantifications (PCC, M_1,2_ and PMC) of biomolecular association as a function of SNR. PCC and M_1,2_ both predictably decrease substantially as SNR decreases. PMC_1_, in contrast, remains comparatively stable over the same range. However, PMC_2_ significantly increases as SNR approaches unreasonable levels. This diverging behavior can act as an indicator of when image quality degrades. *N*=3 samples, error bars indicate standard error.

Pearson's and Manders' coefficients are known to be spuriously affected by image SNR (Aaron et al., 2018; Dunn et al., 2011; Saccenti et al., 2020). This is particularly problematic when dealing with dim and photosensitive samples where compromises in imaging parameters are typically necessary. To examine the susceptibility of the PMC to low SNR, we again labeled MHC with AF488 and its phosphorylated RLC with AF568 in PtK2 cells. We imaged these samples over an order of magnitude range of laser powers (Fig. 7A, low power on the left, high power on the right).  In tandem, we imaged single-color control samples to account for the broadening of the mixing band with worsening SNR (Fig. S6).

Fig. 7C highlights the susceptibility of PCC and M_1,2_ to the SNR. Both coefficients diminished ∼10% in response to a decrease in SNR from 10.5 to 6.0 (Fig. 7C). The PMC, however, indicates greater robustness within this range. PMC_1_, in particular, remained relatively stable against this nearly twofold drop in SNR (Fig. 7C). This is underscored by the relative similarity between the CMIs at low (Fig. 7B, left) and high (Fig. 7B, right) laser power. However, under very low SNR (2.0), PCC and M_1,2_ predictably decreased by a further 10–20% (Fig. 7C). Although PMC_1_ remained comparatively stable at this condition, PMC_2_ deviated by ∼30% (Fig. 7C). This highlights an important limitation. Whereas the average extent of mixing (PMC_1_) is robust to noise, the variation in mixing (PMC_2_) is not. The precipitous changes of PMC_2_ can be a good reminder that rigorous quantitative analysis cannot remedy poor image quality. Nevertheless, over more typical imaging conditions, both PMC values displayed a marked stability against SNR fluctuations compared to PCC and M_1,2_.

### The PMC facilitates colocalization analysis in samples with high background

Background signals, such as autofluorescence, can prevent accurate colocalization analysis (Dunn et al., 2011). To tackle this problem, a common solution is to utilize multispectral imaging and spectral unmixing to separate fluorescence signals (Dickinson et al., 2001; Lansford et al., 2001; Tsurui et al., 2000). Since PMC is based on multispectral imaging, it can be used in conjunction with these unmixing techniques. As a result, we can overcome this persistent issue and quantify color mixing in difficult samples with broad, pervasive background fluorescence.

**Fig. 8.**
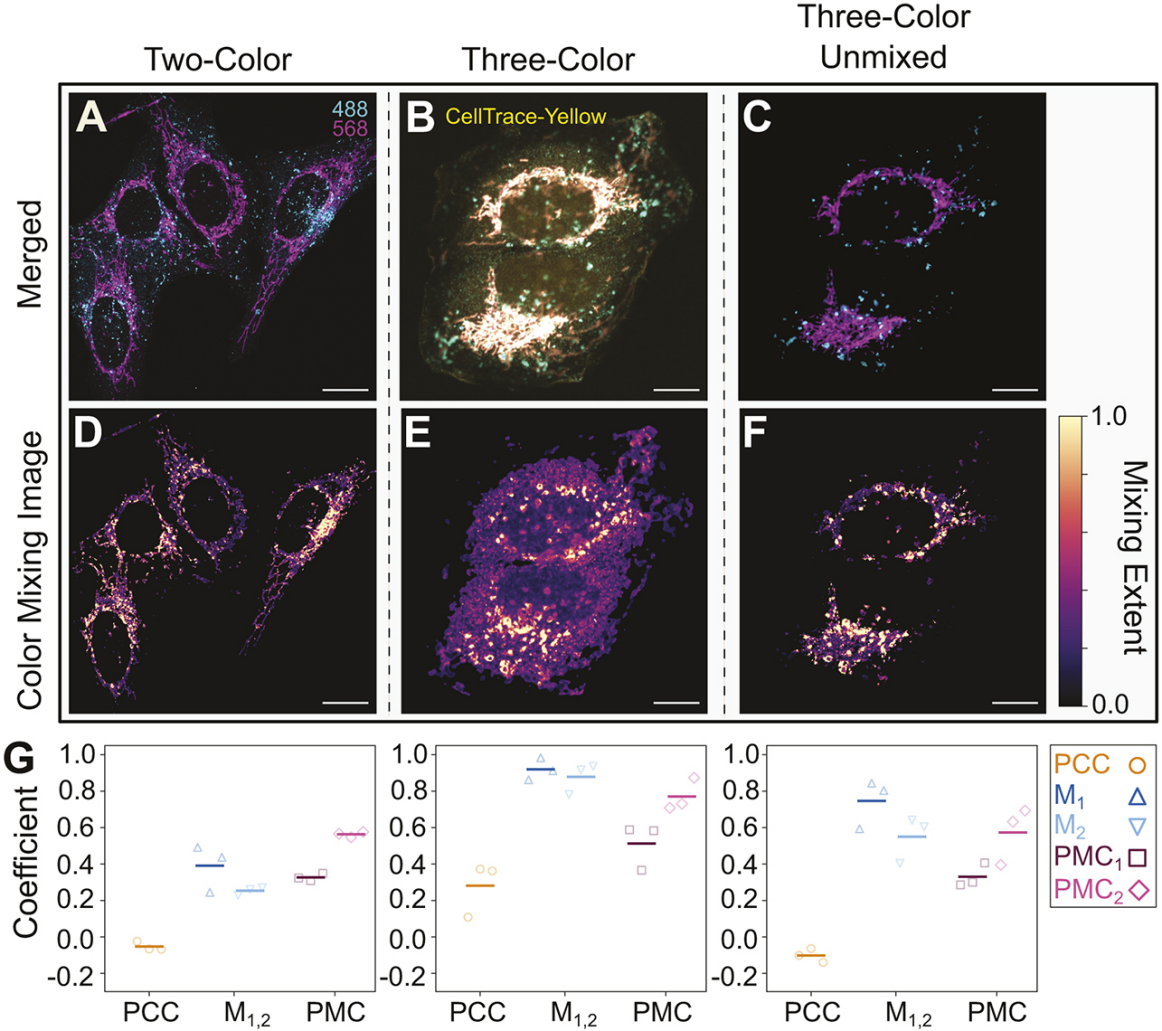
**The PMC facilitates color mixing analysis in samples that exhibit substantial overlapping background signal.** (A) Pseudo-color merged image (extracted from multispectral images) of U2OS cells labeled for LAMP1 (AF488, cyan) and TOMM20 (AF568, magenta). (B) Pseudo-color merged image (extracted from multispectral images) of U2OS cells labeled for LAMP1 (AF488, cyan), TOMM20 (AF568, magenta) and CellTrace Yellow (yellow). Given the significant spectral overlap of CellTrace Yellow and AF568, the regions near the mitochondria appear saturated. (C) Unmixed, pseudo-color merged image of the sample in B. Note the decreased saturation in and around the mitochondria. (D–F) CMIs for the samples in A–C. To highlight the low-intensity regions of mixing caused by CellTrace Yellow, here we employed a one-dimensional LUT as described in the Materials and Methods (also see Fig. S3). In this case, the colormap (shown to the right of F) reflects the product of *f*_*mix*_ and the normalized total intensity. Scale bars: 10 µm. (G) PCC, M_1,2_ and PMC_1,2_ measured for the samples in A–C (from left to right). As expected, the addition of the diffuse CellTrace Yellow increases all measures of mixing (compare left and middle plots). However, using linear unmixing and phasor analysis, the PMC recapitulates the mixing quantification from A in the three-color sample (compare left and right plots). Individual *N*=3 points correspond to individual samples; line marker indicates the mean.

To illustrate this, we first labeled LAMP1 (a marker for lysosomes and exosomes) and TOMM20 (a marker for mitochondria) in U2OS cells with AF488 and AF568, respectively. Unsurprisingly, these structures associated minimally, as quantified by the PMC (PMC_1,2_=[0.33,0.56]), PCC (−0.05) and M_1,2_ (M_1,2_=[0.39,0.25]) (Fig. 8A,D and G, left). We next introduced a third component by incubating cells with CellTrace Yellow, producing a diffuse cytoplasmic background (Fig. 8B,E). This led to an erroneous increase in the association of LAMP1 and TOMM20, with the PCC (0.28), M_1,2_ (M_1,2_=[0.87,0.51]) and PMC (PMC_1,2_=[0.51,0.77]) all increasing (Fig. 8G, middle), as expected.

By integrating spectral unmixing and the phasor method, however, we were able to recapitulate the mixing measurements from the two-color control sample (see Materials and Methods; Fig. S7). We found a relatively low amount of mixing (PMC_1,2_=[0.33,0.57]), consistent with the two-color sample (Fig. 8C,F and G, right). The effect of removing the CellTrace Yellow signal was clear when comparing the CMIs before and after this process (Fig. 8E and F, respectively). Surprisingly, whereas both the PMC and PCC recovered to their control values once the background signal was removed, M_1,2_ did not (M_1,2_=[0.75,0.55]) (Fig. 8G, right). This indicated its susceptibility to image processing. In contrast, this discrepancy further highlighted the robustness of the PMC to confounding experimental factors.

Overall, we took advantage of multispectral imaging to overcome what would otherwise be insurmountable in many colocalization measurements. As the background signal was spectrally distinct from the signals of interest, we could systematically remove its effect. The PMC not only offers nuanced, robust information on color mixing, it also offers the user increased flexibility in their experimental design.

## DISCUSSION

A common first step toward probing the association of two biological objects is to determine whether they colocalize (Adler and Parmryd, 2013). However, colocalization, as we have previously pointed out (Aaron and Chew, 2018), is a misnomer because no two objects can ever occupy the same physical space. To approximate the degree of ‘colocalization’, indirect and often insufficient methods such as correlation and overlap coefficients have been the only tools available thus far. These methods require multiple imaging conditions to be satisfied: the emission of each fluorescent dye must be spectrally distinct, the SNR must be relatively high and the samples must lack any significant background (autofluorescence). Failure to satisfy these constraints will lead to misleading readouts. To bypass these limitations, we reframe the concept of colocalization into one of color mixing. This approach provides a more nuanced representation of biological complexity.

Using phasor-based color mixing analysis, we show that the PMC is more robust than Manders' and Pearson's coefficients against choice of threshold method and background signal. It is also more robust to decreases in SNR over typical ranges, although PMC_2_ can deviate at particularly low SNR. Overall, the PMC better shields its users from erroneous conclusions. Furthermore, it offers greater flexibility when choosing imaging parameters and sample preparation techniques. The PMC method not only quantifies both the extent and homogeneity of color mixing; it also provides an innate ability to visualize spatial differences in color mixing. These features are noticeably missing from Manders' and Pearson's methods, yet are indispensable for biologists to interpret the measurements with critical spatial specificity. Overall, the PMC offers users an analytical environment for probing biological association from multiple angles.

Phasor-based methods such as the PMC can be extended to more complex biological questions. While we focused here on several illustrative cases, colocalization studies are often comparative in nature, such as across different experimental conditions. The range of examples included here demonstrates that the PMC can distinguish and report even subtle changes induced by such perturbations. Broadening this capacity, multispectral imaging naturally lends itself to multiplexed experiments (Garini et al., 2006; Schröck et al., 1996). The powerful properties of phasor analysis are equally applicable to samples with numerous labels (Fereidouni et al., 2012; Torrado et al., 2022; Vallmitjana et al., 2022). Accordingly, the PMC could be adapted to facilitate analysis of more than two colors. The extension of the PMC to an arbitrary number of components is an area of future work. Moreover, the inherent precision with which phasor analysis can distinguish emission spectra opens the door to probing the mixing of colors with very similar spectra. This would prove especially useful in situations where the palette of available labels is prohibitively narrow. In general, phasor-based mixing analysis offers the user more flexibility and expands the range of potential experiments.

Notably, the use of phasor-based analysis necessitates multispectral imaging, which might involve additional considerations. It requires microscopy hardware that is available on most modern laser scanning confocal microscopes. The specific hardware will determine the spectral resolution of the system. It has been shown that as few as four spectral channels are needed to perform phasor analysis (Hedde et al., 2021; Longo et al., 2025; Torrado et al., 2022). However, the optimal number of spectral channels will be highly dependent on the spectral characteristics of the fluorophores being imaged. Moreover, without specialized detectors, spectral imaging can be too slow for live-cell imaging. This limitation can be assuaged by multi-element detectors capable of fast multispectral imaging. Nonetheless, a researcher should always determine the minimum temporal resolution needed to accurately capture the biological event, and whether their multispectral instrument can deliver that acquisition speed. Spectral imaging will reveal distinct artifacts in the form of motion-induced streaking throughout the lambda stack when the temporal resolution is insufficient (Hedde et al., 2021; Parker et al., 2023). Thus, applying the PMC method to live-cell imaging will depend on the available imaging system. Importantly, the PMC method can be used on any system that satisfies the spectral resolution requirements highlighted above. The addition of multiple channels and filters to any system would therefore facilitate PMC analysis accordingly.

It is important to note, however, that the accuracy of PMC is diminished by intensity imbalances between each color component to a greater degree than M_1,2_ and PCC (see Fig. S8). As we and others have shown, the contribution of each species reflects the number of photons emitted by each species at each pixel (Malacrida et al., 2021; Ranjit et al., 2019; Torrado et al., 2022). This does not discriminate between two possible reasons for a smaller fractional component of one signal: a smaller abundance of fluorophores or simply dimmer fluorescence. This reflects the sensitivity of the method but also represents a known and significant consideration of multispectral imaging and phasor analysis (Malacrida et al., 2021). Nevertheless, using fluorophores of comparable brightness and optimizing imaging parameters with this in mind will ameliorate this issue.

Fundamentally, the measurement of how two objects associate with one another is determined by the optical resolution. This remains true when employing multispectral imaging. A pixel located in the region where two objects overlap will have a mixture of two spectra. It is important to note that this spectral information cannot be conveniently separated through computational means such as spatial deconvolution. It is imperative, therefore, that users judiciously select the microscope appropriate for the biological question (Aaron et al., 2019; Wait et al., 2020). However, as has been shown here and elsewhere, biological quantification methods can be insidiously susceptible to experimental choice (Aaron et al., 2018; Bolte and Cordelières, 2006; Costes et al., 2004; Dunn et al., 2011). More fundamentally, however, they often fail to sufficiently describe the underlying biology, such as we have shown here. Ultimately, in a literature replete with convention and conflicting recommendations, users should carefully choose the method that will best inform their central biological question.

## MATERIALS AND METHODS

### Sample preparation

#### Sample preparation for data shown in Fig. 5A,C

U2OS cells (Sigma, 92022711-1VL) were grown in McCoys's 5A (Gibco, 16600-082) supplemented with 10% FBS (ATCC, 30-2020), at 37°C in 5% CO_2_, to 70% confluency. Cells were quickly rinsed three times with PBS, pH 7.4 (Gibco, 10010023), then dissociated using 0.25% trypsin 0.53 mM EDTA solution (Fisher, 25-200-056). Cells were checked for detachment every minute. Then cells were spun down at 800 ***g*** for four minutes, and the supernatant was discarded. Cells were resuspended in fresh growth medium and seeded on 35 mm MatTek dishes with no. 1.5 coverslips (MatTek, P35G-1.5-14-C) at 1:20 dilution, and grown for 48–72 h prior to fixation.

Samples were fixed in pre-warmed (37°C) 4% paraformaldehyde (Thermo Scientific, 28906) in 1× PBS (Gibco, 10010023) for 10 min. This, and all subsequent steps were performed at room temperature, on a rocking platform, unless otherwise noted. Three washes in 1× PBS were performed for 5 min each, on a rocking platform. Cells were permeabilized with 0.3% v/v Triton X-100 (Sigma, T8787) in 1× PBS for 10 min, rocking, followed by three more washes in 1× PBS, 5 min each. Cells were blocked for 60 min in blocking buffer: 2% BSA (Sigma, A2153), 22.52 mg/ml glycine (Sigma, G7126), 1× PBS, 0.1% Tween20 (Sigma Aldrich, P7949). Rabbit IgG anti-TOMM20 (eLabScience, E-AB-81488) was diluted to 300 ng/ml in blocking buffer, and slides were incubated at 4°C in a humid, dark chamber overnight. The next day, samples were washed with 1× PBS three times, 5 min each. For Fig. 5A, two secondary antibodies were used: goat anti-rabbit IgG–AF488 (Invitrogen, A-11008) and goat anti-rabbit IgG–AF555 (Invitrogen, A21430). For Fig. 5C, goat anti-rabbit IgG–AF488 was used in conjunction with 3 μM Hoechst–JF549 (kind gift from Luke Lavis, Howard Hughes Medical Institute, Janelia Research Campus, VA, USA). In each case, the AF-conjugated secondary antibodies were diluted in blocking buffer to each be at a concentration of 2 μg/ml and incubated on slides for 60 min. Slides were washed three times, five minutes each, with 1× PBS. Coverslips were mounted to slides using Prolong Gold (Invitrogen, P10144) and stored in the dark at room temperature for 24 h to cure before being stored at 4°C until imaging.

#### Sample preparation for data shown in Figs 5B, 6 and 7

PtK2 cells (NBL-5; ATCC CCL-56) were grown to 80% confluency in MEM (Corning 10-010-CV) supplemented with non-essential amino acids (Sigma Aldrich, M7145), sodium pyruvate (Gibco, 11-360-070) and 10% FBS (ATCC, 30-2020), at 37°C in 5% CO_2_. Cells were washed three times with 1× PBS (Gibco, 10010023), then dissociated with 2 ml of 0.25% trypsin 0.53 mM EDTA solution (Fisher, 25-200-056), checking for detachment every minute. Once cells were detached, they were dispersed with 10 ml of pre-warmed (37°C) growth medium and spun down at 1000 ***g*** for 8 min. Supernatant was discarded and cells were resuspended in fresh growth medium. Cells were seeded onto 22 mm×22 mm coverslips (Fisher, 12541016; no. 1.5) at 1:10 dilution and grown for 24 h before fixation.

Cells were fixed in pre-warmed (37°C) 3.7% formaldehyde in PBS, pH 7.4 for 5 min at room temperature. Permeabilization was accomplished using 0.2% Triton X-100 (Sigma, T8787) in 1× PBS for 2 min. Coverslips were washed in 1× PBS for 5 min. Mouse anti-myosin heavy chain, IgM, cl. H11 (BioLegend, 922701; to detect MHC) and rabbit anti-pMyosin (Cell Signaling Technology, 3674S; to detect phosphorylated RLC) were used at 1:200 dilution in 1× PBS containing 1% BSA (Sigma, A2153). Primary antibodies were incubated for 1 h at 37°C. Cells were washed with 1× PBS three times, then secondary antibodies were added: goat anti-rabbit IgG–AF568 (Life Technologies, A110011) diluted to 1:25 and goat anti-mouse IgM–AF488 (Fisher, A21042) diluted to 1:400 in 1% BSA in 1× PBS. Cells were again incubated for 1 h at 37°C, then rinsed with 1× PBS. Coverslips were mounted on Prolong Gold (Invitrogen, P10144). Control sample was stained only with either rabbit anti-pMyosin (goat anti-rabbit IgG–AF568) or mouse anti-myosin heavy chain (goat anti-mouse IgM–AF488).

#### Sample preparation for data shown in Fig. 8

U2OS cells (Sigma, 92022711-1VL) were grown in McCoy's 5A (Gibco, 16600-082) supplemented with 10% FBS (ATCC, 30-2020) and 2 mM L-glutamine (Gibco, 25030081) in an incubator maintained at 5% CO_2_ and 37°C. Cells were dissociated using 0.05% trypsin–EDTA (Gibco, 25300054) and plated on no. 1.5 glass coverslips. After 24 h, cells were stained for 20 min with 5 μM CellTrace Yellow (Invitrogen, C34573A) in OptiMEM (Gibco, 31985070), followed by two washes in PBS, and then left in growth medium for 20 min before fixation. For the samples lacking CellTrace Yellow, these steps were omitted accordingly.

After three quick washes in warm (37°C) PBS (Gibco, 10010023), samples were fixed. Fixation was done using a solution pre-warmed to 37°C containing 0.1% glutaraldehyde (EMS, 16019) and 3% formaldehyde (Thermo Scientific, 28906) in PBS, for 5 min at room temperature. Permeabilization was done using 0.1% Triton X-100 (Sigma, T8787) for 5 min at room temperature. Reduction of glutaraldehyde was done using 1 mg/ml sodium borohydride (Sigma, 213462) for 7.5 min at room temperature. Fixation, permeabilization and reduction steps were all followed by three quick washes in PBS.

Cells were stained with rabbit anti-TOMM20 (eLabScience, E-AB-81488) at 1:500 and mouse anti-LAMP1 (eBioScience, 14-1079-80) at 1:200 in PBS including 0.1 mg/ml IgG-free bovine serum albumin. Primary antibody staining was done for 45 min at 37°C. Then, 3×5 min washes were done in PBS. Cells were then stained with goat-anti-rabbit IgG–AF568 (Invitrogen, A11011) at 1:25 and goat-anti-mouse IgG–AF488 (Invitrogen, A11001) at 1:500 in PBS with 0.1 mg/ml IgG-free bovine serum albumin. Secondary antibody staining was done for 45 min at 37°C. Then, 3×5 min washes were done in PBS. Lastly, coverslips were mounted to slides using Prolong Gold Antifade Reagent (Invitrogen, P10144). The sample was kept at room temperature in the dark to cure for 24 h before imaging.

### Multispectral imaging

All images were acquired as 16-bit images using a Zeiss LSM 980 inverted laser scanning confocal microscopy, equipped with a 32-channel GaAsP multi-PMT array for spectral detection. All images were acquired using a Zeiss 63× Plan-Apochromat objective with a 1.40 NA. All images were acquired at a single *z*-plane at Nyquist sampling.

For multispectral detection, samples were simultaneously illuminated with 488 nm and 561 nm lasers with typical powers ranging from 0.1% to 2%. Most images were acquired using unidirectional scanning, with spectral detection range of 500–690 nm, using bins of 8.8 nm. The images for Fig. 8 were acquired using a spectral range of 412–690 nm, also with bins of 8.8 nm. A dual-bandpass 488 nm/561 nm dichroic was used to block excitation light from the detector. Images were typically acquired using a gain of 600–750 V and a positive offset of 0–1000. For specific parameters corresponding to each figure, please see Tables S1–S5.

### Computational methods

Analysis code can be found in the associated repository (https://github.com/aicjanelia/phasor_mixing). All computations were conducted in Python (v3.10.12) with the corresponding libraries: numpy, scikit-learn, scikit-image and scipy. Plots were made using the Python library matplotlib. The minimal required packages can be installed using the phasor_requirements.txt file provided in the repository and the environment manager of choice.

All computations were performed on a Windows 11 personal computer running Windows Subsystem for Linux (WSL-Ubuntu) equipped with an Intel^®^ Xeon^®^ w7-3465X processor and 512 GB RAM. All computations were performed on the CPU. No GPU is necessary.

#### Simulating multispectral images

For Figs 1 and 4, we simulated intensity and multispectral images from artificial single-color intensity images. The artificial intensity images (Fig. 1) were created using the graphics editor Inkscape (https://inkscape.org/) and scaled to 8-bit range. Without modification, these are taken as single-color intensity images for the purposes of display. For Fig. 1 (as throughout), Pearson's and Manders' coefficients were calculated as described in ‘Calculating PCC and M_1,2_ from multispectral images’ below.

To simulate multispectral images from these single-color intensity inputs, we first identified spectra, in this case for AF561 and AF647. The spectra were converted to probabilities by dividing each spectrum by its summed intensity. For a given number of photons, we randomly drew an ‘emission’ from this spectral probability function, creating a distribution of photons emitted at each wavelength, for each pixel. Gaussian background noise was added to this distribution. The resulting distribution was then binned to simulate the spectral resolution (32 gates of size ∼9 nm) of the microscope. Finally, these binned signals were multiplied by the intensity of the input images. This was repeated for each simulated signal. These multispectral images were then added together element-wise so that the final image contains information from each signal at each wavelength.

#### Phasor methods

##### The phasor transform

Phasor analysis transforms a spectrum into a single point. This is done by displaying the width and position of the decay or spectrum in the form of phasor coordinates (*G*, *S*) (Weber, 1981). These coordinates are calculated pixel-wise from the collected emission spectra (Eqn 3):
(3)

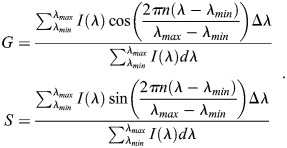
This transformation is based on the Fourier transform (Digman et al., 2008; Fereidouni et al., 2012; Weber, 1981). These coordinates are bounded by the unit circle – i.e. (*G*, *S*) ∈ [−1,1] – and are unitless. One can also rewrite these coordinates in terms of the polar coordinates (*m*, ϕ) (Eqn 4):
(4)

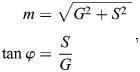
where ϕ represents the center of mass of the emission spectra, and *m* represents the full-width at half-max (FWHM) of the spectra.

Sets of pixels containing a single fluorophore will be mapped to a phasor cluster of some width that is related to the SNR: the radius of the cluster 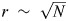, where *N* is the number of photons detected (Cutrale et al., 2017; Leben et al., 2019; Ranjit et al., 2018a). Phasor coordinates were subsequently smoothed by applying an optional median filter of radius three, as is commonly applied (Ranjit et al., 2018b). This filtering was not performed on the data for Fig. 7 as the potential effects of noise were under direct inspection.

##### Clustering to determine pure components

For each pure component image, we need to compute a reference point for the phasor cluster: we define this to be the centroid of the phasor cluster. We calculate this point through a two-step clustering process using scikit-learn implementations (see Fig. S1). Both clustering steps operate first on a small test set of data, and then the remaining data are assigned using an inductive classifier.

First, the entire phasor cluster – every pixel – is separated into *n* clusters using Gaussian mixture models (GMMs): *n*−1 background (to account for potential non-specific staining) and one rough pure component. However, GMMs assume elliptical clusters and strictly assign each point to a cluster. Since the background cluster is not elliptical, this results in some background pixels being assigned to the pure component cluster. As a result, we refine the pure component cluster using a second step of hierarchical density-based spatial clustering of applications with noise (HDBSCAN). This method is not strict and thus leaves the remaining background pixels as outliers (not assigned). Both steps involve minimal parameter tuning – the number of clusters (*n*=2) in the case of GMM, and the minimum number of points that define a cluster in the case of HDBSCAN. Otherwise, the default parameters of the scikit-learn implementation of these methods were used, and in the case of HDBSCAN, the single non-default parameter was held constant for each application throughout this study.

We estimate the density of the resulting cluster at every point using kernel density estimation (KDE). The pure component centroid is defined to be the point of maximum density. This is determined by evaluating the KDE model at a uniform set of points across the bounding region of the fine-tuned cluster. The peak value of this evaluated density is taken as the centroid. This process is repeated for the number of pure components expected in the multicolor sample.

##### Defining the mixing band

Pixels containing two signals are mapped to phasor points along the line connecting the two pure components. To restrict the analysis to only points along this line, we define a mixing band of some thickness around this line (to account for finite noise). Instead of arbitrarily choosing this thickness, we instead use a data-informed approach by leveraging the fact that the size of the pure component clusters reflects the SNR of the system.

For each pure component cluster, we use principal component analysis (PCA) to decompose the cluster of points into two components. We then calculate the maximum explained variance (eigenvalue of the covariance matrix, 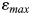) across the group of pure component clusters. We set the band thickness (or threshold) to be a multiple (1.96 by default) of this maximum. This default value was used throughout all analysis presented. We will call this value the ‘threshold’*.* The vertices of this band ultimately define a rectangular region in phasor space which contains the phasor points to be considered for further analysis. We will refer to the phasor cluster within this region as the ‘mixing cluster’*.*

##### Component analysis

To calculate the contribution of each pure component in each pixel, we utilize the law of phasor addition (Torrado et al., 2022). Given the points inside the mixing band, we first project each phasor point onto the line connecting the two pure components using standard vector projection. Then, for each projected point (*G*, *S*), we calculate the fraction *f*_*i*_ of each component by solving the linear system of equations using the non-negative least squares (Eqn 5):
(5)

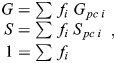
where *f*_*i*_ ∈ [0, 1]. For systems of two components (three components is handled separately below), we then define a new quantity (Eqn 6):
(6)

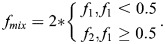
This accounts for the symmetry around ‘perfect mixing’ of *f*_1_=*f*_2_=0.5.

##### The phasor mixing coefficient

The PMC contains two separate quantities. *µ*=PMC_1_ is defined as the expected value of *f*_*mix*_. The probability density function *K* is estimated using kernel density estimation. Then (Eqn 7):
(7)

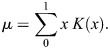
The second quantity of the PMC is defined as a normalized size (or variance) of the mixing cluster. This size is normalized by two factors: the size of an individual pure component phasor cluster, and the size of both pure component clusters concatenated. As explained in the ‘Defining the mixing band’ section above*,* we calculate the size of a pure component cluster by taking the maximum eigenvalue of the covariance matrix as determined by PCA. Let *σ*_*PC*_ be the maximum pure component size across all pure components. Next, we concatenate the two pure component phasor clusters whose mixing we wish to quantify. For this combined phasor cluster, we again calculate the maximum eigenvalue of the covariance matrix (again via PCA). Let this be *σ*_*max*_. We reasoned that the mixing cluster would be ‘largest’ if it corresponded to two entirely separated signals. These two quantities set the bounds on the size of the mixing cluster. Finally, we measure the size of the mixing cluster using the same method to obtain *σ*_*mix*_. This allows us to calculate PMC_2_=*σ* (Eqn 8):
(8)

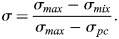
Taken together, [*μ*, *σ*]=[PMC_1_, PMC_2_]≡PMC. We acknowledge that choosing these bounds results in the situation where spatial exclusion corresponds to zero variance. While this may be counterintuitive, this situation is easily discerned through PMC_1_ and biological reasoning. Moreover, choosing this scaling allows the user to discriminate other, more subtle situations, as shown.

##### The color mixing image

To quantitatively display the mixing extent in every pixel of the multispectral image, we create a color mixing image (CMI). Due to phasor reciprocity, the mixing extent *f*_*mix*_ is defined at every pixel and can be mapped back to image space. To do so, we map the 2D vector of the mixing extent and summed intensity to a 2D lookup table (LUT) where the color represents *f*_*mix*_ and the brightness represents the intensity. This is done by converting the chosen RGB colormap to an HSV colormap. We then map *f*_*mix*_ to the hue (H) and the summed intensity to the value (V) along 8-bit (256) bins. We then convert this HSV image back to RGB for display purposes. Minimum values for the hue and value can be set to saturate either color or brightness, accordingly. In the mixing image, regions of high brightness and color (defined by the chosen colormap) reflect an abundance of highly mixed signal; high brightness and low color regions, conversely, reflect low mixing; and low brightness regions reflect low overall signal. Colormaps were chosen from either matplotlib or the library of perceptually uniform colormaps from the python package colorcet, as labeled. Users can choose any colormap from these resources by specifying its name where indicated in the code.

A separate method is available in which the product of *f*_*mix*_ and the normalized summed intensity is mapped to a one-dimensional LUT (simply the colormap in this case). This method displays the combined mixing and intensity information on one scale, as shown in Fig. 8 and Fig. S3.

#### Calculating PCC and M_1,2_ from multispectral images

To approximate single-color intensity images from multispectral images, we first identify the spectral peaks for each pure component. For each pure component image, we average over all foreground pixels in *x*-*y* to obtain a mean spectral signal. We then use scipy to find the location (wavelength) of the most prominent peak of this signal. We then select the three λ-slices surrounding this peak, including the peak slice itself. Summing these three slices over wavelength, we obtain an approximate ‘single-color’ pure component intensity image. Using these intensity images and any specified intensity-based thresholding method, PCC and M_1,2_ are calculated as normal, using the scikit-image implementations of each. Unless otherwise specified, PCC and M_1,2_ were calculated after applying intensity thresholds determined using the Otsu method.

#### Comparing PCC, M_1,2_ and PMC across SNR ranges

For comparing coefficients across ranges of SNR, we use 
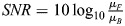
, where *μ*_*F*_ and *μ*_*B*_ are the mean summed intensity of all foreground and background pixels, respectively. In this case, no intensity thresholds were applied at any step, to fully illustrate the effect of poor SNR.

#### Handling background signal

##### Image processing

Due to significant non-specific staining of the LAMP1 in these samples, we applied intensity thresholding to mitigate this effect. For the samples without CellTrace Yellow, we applied the ISODATA method to calculate the threshold. We also utilized this method for calculating PCC and M_1,2_ from the images after the background signal was removed using the phasor method. Additionally, in the three-color sample before CellTrace Yellow signal was removed, we applied the Triangle method to calculate a threshold that successfully masked the entire cell area. Due to the low overall intensity of the CellTrace Yellow, other thresholding methods tended to mask out some of its effect. We intentionally chose a method that would highlight the worst-case scenario.

##### Linear unmixing pure component images

Single-color multispectral images were acquired as described above. Using the Zen software, we use Automatic Component Extraction (ACE) to isolate spectra for both the autofluorescence [*a*(*λ*)] and the fluorophore signal [*b*(*λ*)]. With these approximated spectra and the multispectral image, we used non-negative least squares to solve for the relative contributions (*A*_*i*,*j*_, *B*_*i*,*j*_) of the autofluorescence and fluorophore in each pixel *p*_*i*,*j*_ (Eqn 9):
(9)

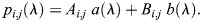
Using this, we take the outer product of the autofluorescence intensity image *A* and the autofluorescence spectra *a*(*λ*) and subtract this from the original single-color multispectral image. The result corresponds to the single-color multispectral image without autofluorescence. With this image in hand, the process for determining the pure component is performed as described. This is repeated for each single-color image. Additionally, we image an unlabeled sample to obtain an autofluorescence-only multispectral image. We also identify the centroid of this ‘pure component’, which is used to perform the subsequent three-component analysis.

##### Restricting analysis to the pure component-defined triangle

In samples that contain three pure components (such as a sample with autofluorescence), the relevant pixels are contained within the triangle defined by the three pure component centroids. As before, to account for error in determining the pure components, we use the same threshold (explained above) to magnify the pure component-defined triangle slightly, while keeping the center of the triangle constant. Then, using these scaled vertices, we identify points inside the triangle using the following logic.

Let the scaled triangle vertices be the points A, B and C. Then the edges of the triangle are denoted AB, BC and CA. For each point P, we ask: is P to the left of or to the right of both the lines AB and CA? If it is, then P cannot be inside the triangle. If it is not, then P is at least inside the wedge defined by these two lines. Now since a point inside a triangle must be to the same side of AB as BC (and also CA), we check whether the position of P relative to these edges differs. If they do, P cannot possibly be inside, so we deduce that P sits inside the triangle.

##### Linear unmixing in phasor space

As in the case of only two components, to calculate the fractional contributions of the two fluorophores and background (CellTrace Yellow), we solve Eqn 1 as before (in this case, there are three fractions: *f*_1, 2, 3_). We then recreate a ‘background’ image by taking the autofluorescence fraction (*f*_3_) at each pixel and multiplying it by the mean foreground intensity of the background pure component image. Taking the cross product of this ‘background fraction’ image and the measured background spectra results in a multispectral background image, which is then subtracted from the original multispectral image. This represents the final unmixed multispectral image. The color mixing analysis follows as described above, using this image as input.

## Supplementary Material

10.1242/joces.264388_sup1 Supplementary information
