# Supplementary material for: Redefining colocalization analysis with a novel phasor mixing coefficient
Source: J Cell Sci. 2026 Jan 19;139(1):jcs264388. doi: 10.1242/jcs.264388 (PMC12863294; doi:10.1242/jcs.264388)
Supplement: Supplementary information [file joces-139-264388-s1.pdf]

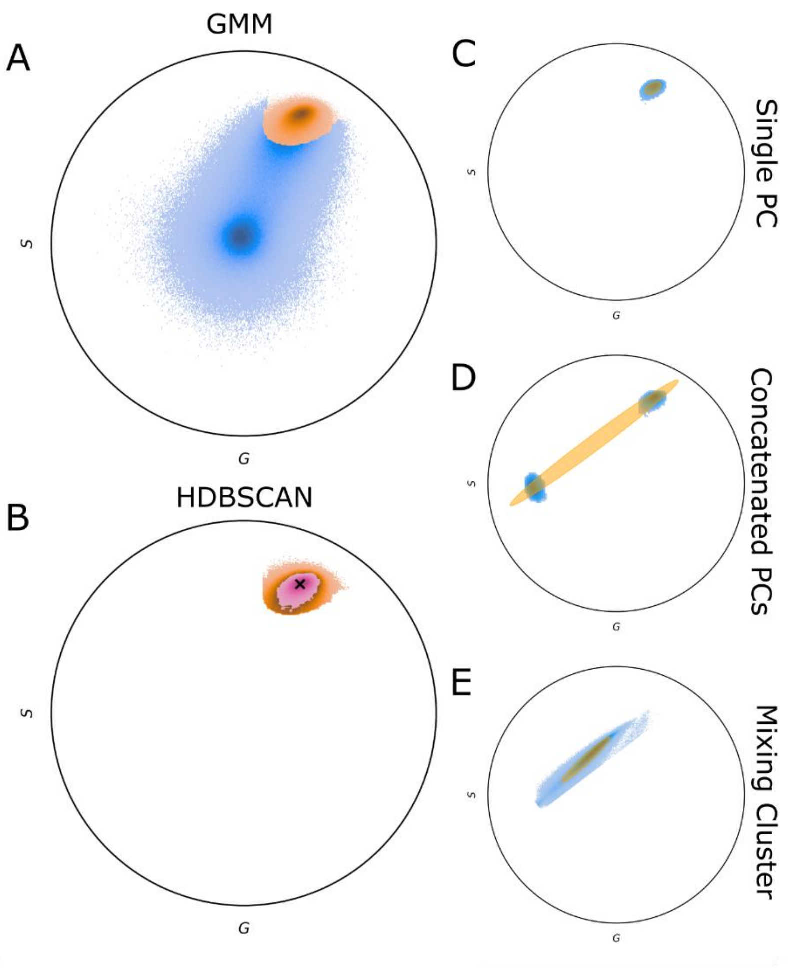

Fig. S1. PC clustering and mixing band definition Pure component (PC) phasor coordinates are determined in a two-step process. A) First, Gaussian Mixture Models (GMM) is used to roughly cluster phasor coordinates into background (blue) and foreground (orange) clusters. B) Second, the rough foreground cluster (orange) is then refined using HDBSCAN into the PC cluster (magenta). The centroid of this cluster (black X) represents the coordinates of the PC. C-E) The size of a phasor cluster is calculated by the taking magnitude of the maximum eigenvalue of its covariance matrix, as determined by PCA. This is reflected in the length of the major axis of the orange ellipse. For the PC cluster (C), this size sets two things: the upper-bound of PMC_2, and the size of the mixing band. D) Similarly, treating the two PC clusters as one single cluster (D), its size determines the lower-bound of PMC_2. The size of the cluster formed by the phasor points within the mixing band (E) is normalized by the two preceding values to form PMC_2.

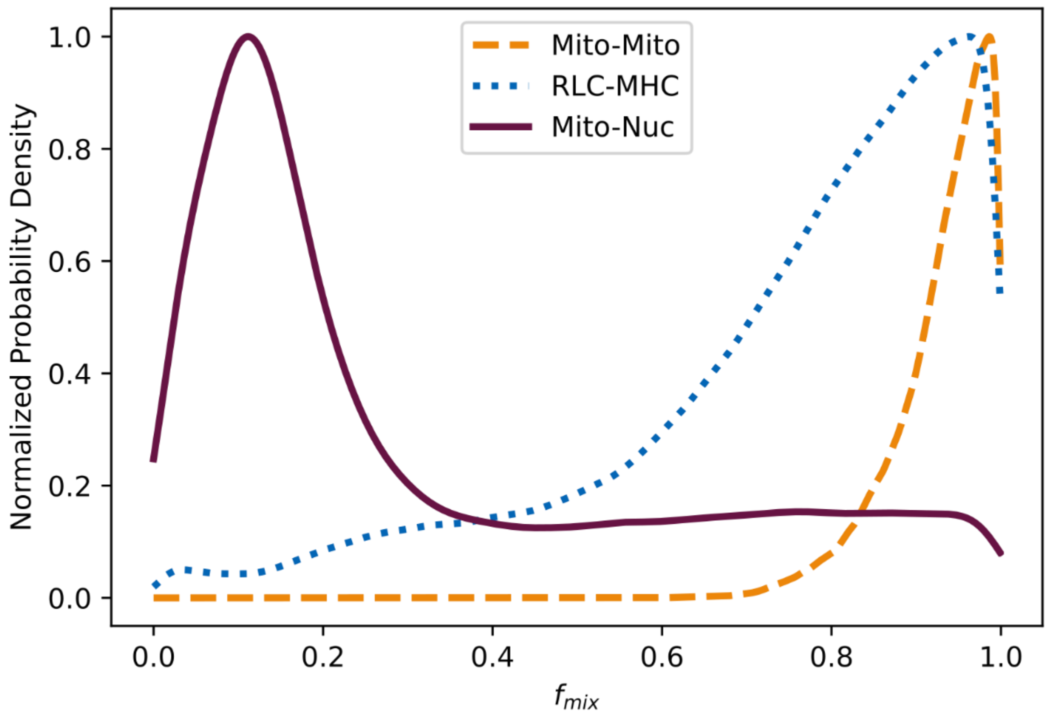

Fig. S2. The mixing distribution Projecting all phasor points within the mixing band onto the line connecting the two PCs results in a distribution we called the Mixing Distribution. This is displayed here for the samples from Fig. 5 in the main text. Comparing the mixing distributions for the Mito-Mito and Myo-pMyo samples, the former exhibits a narrower distribution around 1.0. This reflects its larger PMC_2 value. Similarly, the mixing distribution for the Mito-Nuc sample is narrowly centered near 0.1. This points to its low $PMC_{1,2}$ values in turn.

Journal of Cell Science • Supplementary information

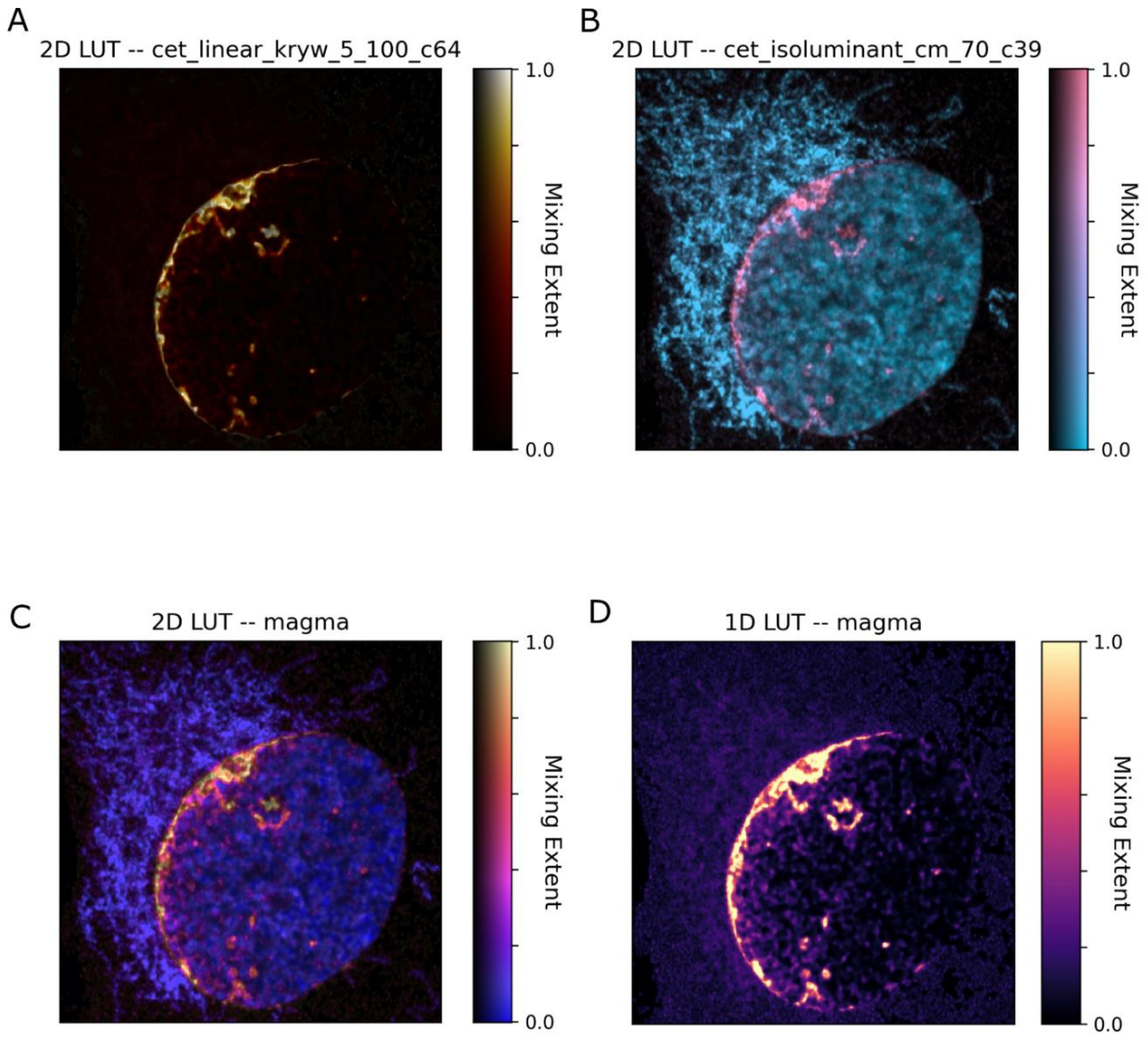

Fig. S3. The Color Mixing Image contains nuanced information that can be visualized by using different LUT's and colormaps Depending on the choice of lookup table (LUT) and colormap, the color mixing image can highlight distinct aspects of signal mixing in the same image. A-C) CMI's produced using a 2D LUT (see Methods) from three different colormaps, with the corresponding names given above. These data were all taken from Fig. 5C. The colormaps in A) and B) were specified from the colorcet Python package, while that in C) is standard in matplotlib. The colormap in A) maps small mixing values (near 0.0) to black. Combined with intensity modulation, this CMI highlights areas of high mixing and high total intensity. Conversely, the colormap in B) maps low mixing values to a non-zero hue. As a result, this CMI highlights the contrast between regions of spatial exclusion (cyan) and 50:50 mixing (magenta). Finally, C) displays the gradation between exclusion and mixing (this is the same CMI as in Fig. 5C). D) Instead, a 1D LUT could also be used. In this case, the colormap reflects the product of f_{mix} and the normalized total intensity at each pixel. This method can be useful to highlight areas of relatively high mixing but low intensity when signal imbalances are significant, such as in Fig. 8E.

Journal of Cell Science • Supplementary information

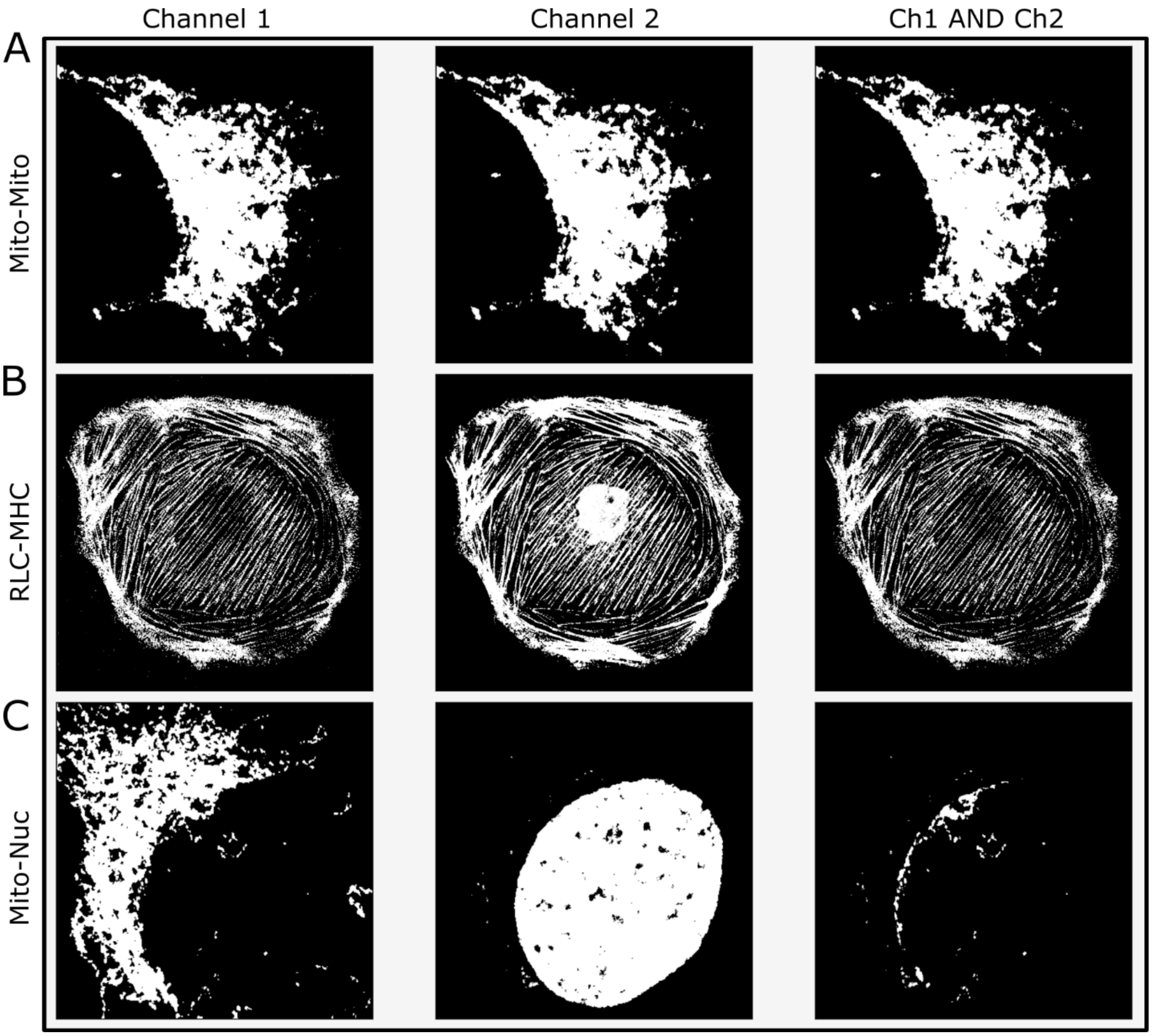

Fig. S4. Representative binary masks for images from Fig. 5 for the purpose of calculating PCC and $M_{1,2}$ A-C) Artificial filter images for each channel were extracted from lambda stacks from each example in Figure 5, as described in the Methods. Otsu's method was then used to determine intensity thresholds in the intensity image for each channel. Binary masks for Channel 1 (left), Channel 2 (middle) were defined by applying the corresponding threshold and were used to calculate $M_{1,2}$ accordingly. To calculate PCC, the logical AND operator was applied to the Ch1 and Ch2 masks to obtain the intersection (right).

Journal of Cell Science • Supplementary information

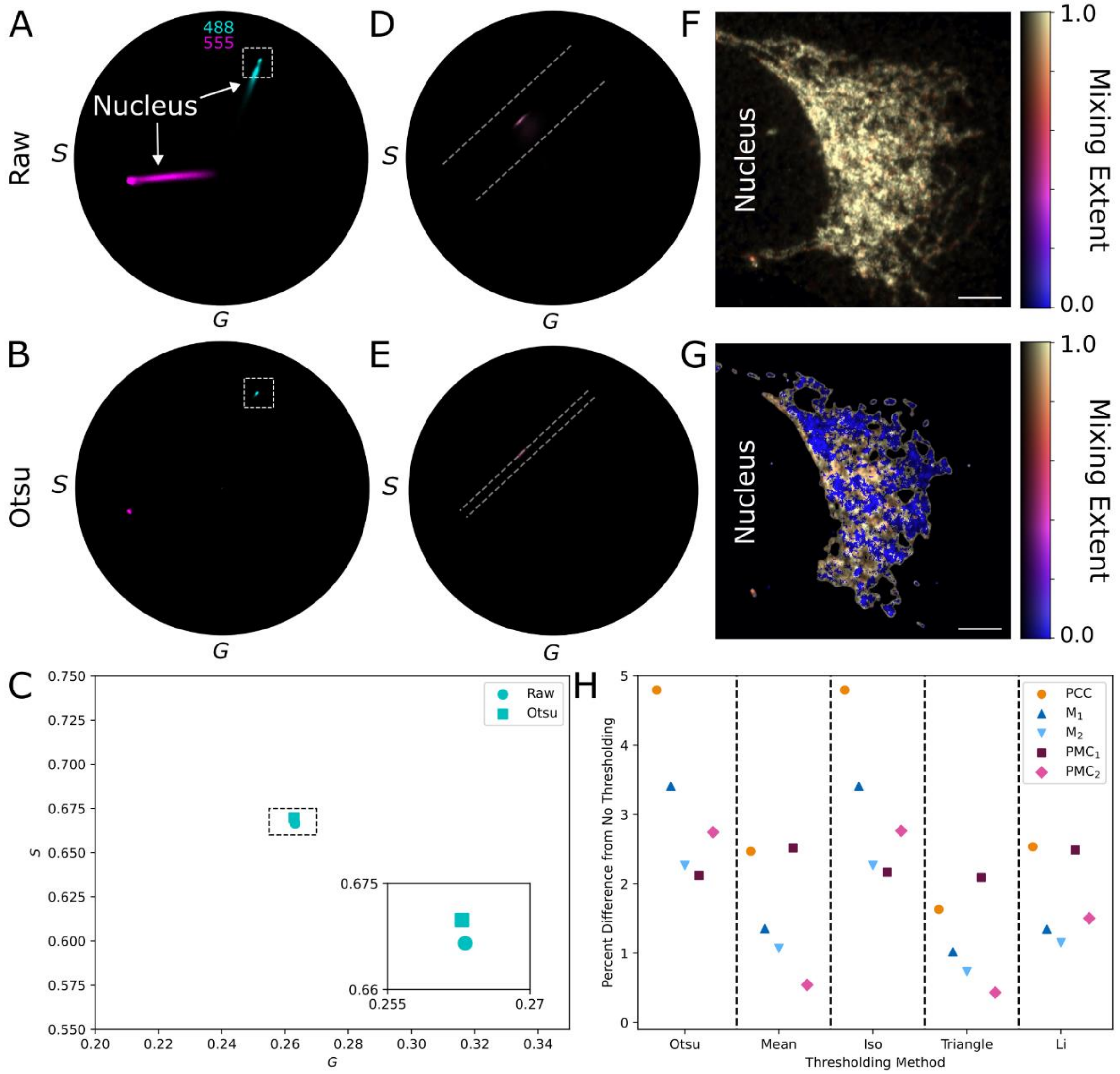

Fig. S5. Thresholding effects in certain situations A) Phasor plots for the pure component images (AF488 and AF555) corresponding to the TOMM20 double-stained sample in Fig. 5A. The long tails in the phasor plots correspond to points within the nucleus that exhibit non-specific staining of low intensity. B) The phasor plot for the same image from A) but with an intensity threshold set by Otsu's method. Note the tails are removed and the size of the phasor cluster is reduced. The dotted boxes in A) and B) correspond to the bounds of the plot in C). C) The centroids of the AF488 phasor plots in A) and B). The inset shows a small difference in their location. D-E) The mixing bands for the double-labeled image in Figure 5A without (D) and with (E) an intensity threshold. The smaller phasor clusters in B) gives rise to a narrower mixing band in E). Note how the bounds of the mixing band intersect the mixed phasor cluster. F-G) Color Mixing Images for the same image without (F) and with (G) the intensity threshold applied. The combination of the intensity threshold itself and the narrow mixing band leads to some exclusion of foreground pixels. In particular, the blue in G) correspond to those excluded by the bounds of the mixing band. While shown here for clarity, these pixels do not factor into the calculation of PMC. Scalebar is 10μm and applies to both F and G. H) The percent difference between the measured coefficient without thresholding and various methods. Regardless of the choice of method, applying an intensity threshold results in a small difference (<5%) for each coefficient.

Journal of Cell Science • Supplementary information

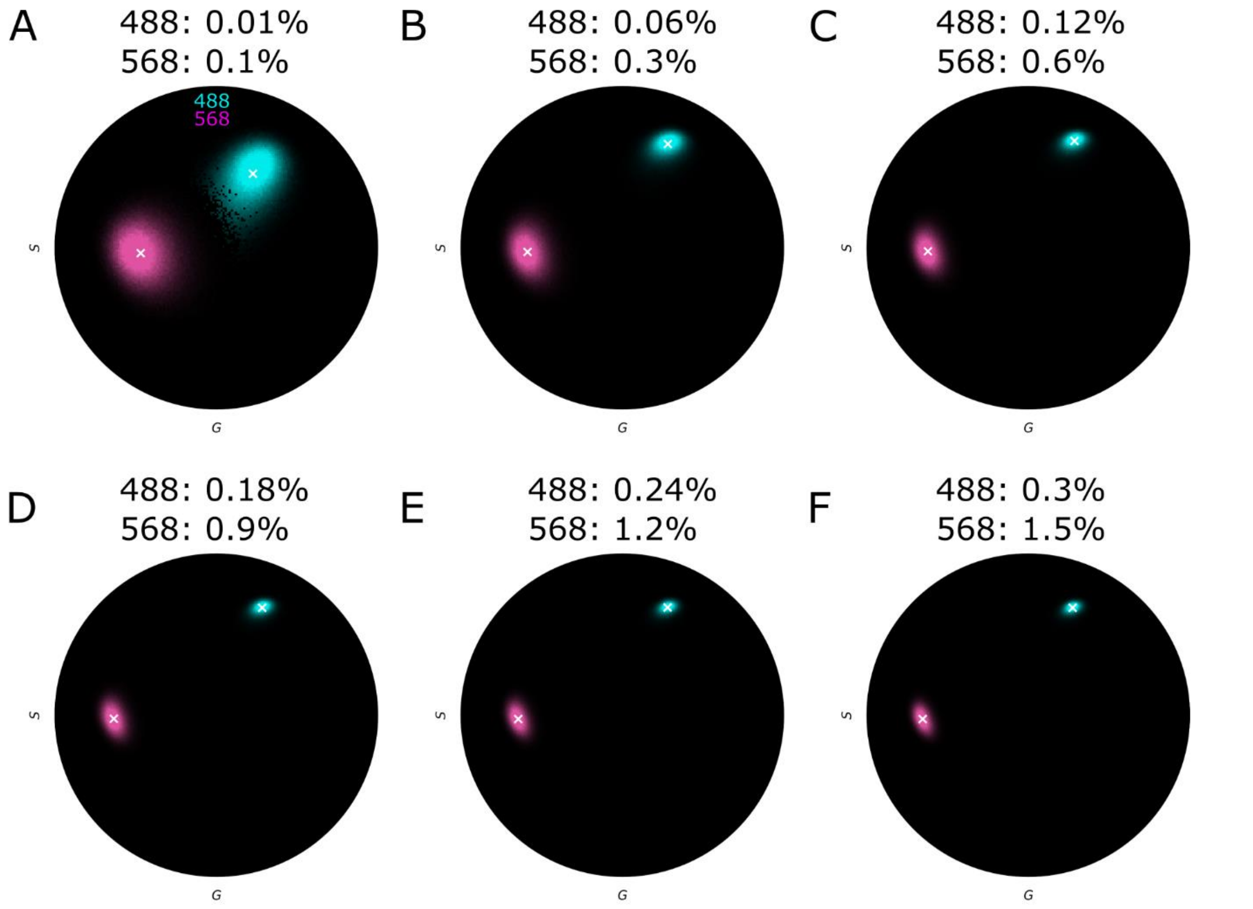

Fig. S6. Effect of SNR on PC cluster size A-F) SNR decreases as one moves right and down (from A to F). The laser power for each fluorophore is given as a header above each phasor plot (given as a percentage for each laser line corresponding to the fluorophore denoted). The white X's indicate the determined PC coordinates. As SNR increases, the size of the PC cluster decreases, and the PC coordinates shift radially outward.

Journal of Cell Science • Supplementary information

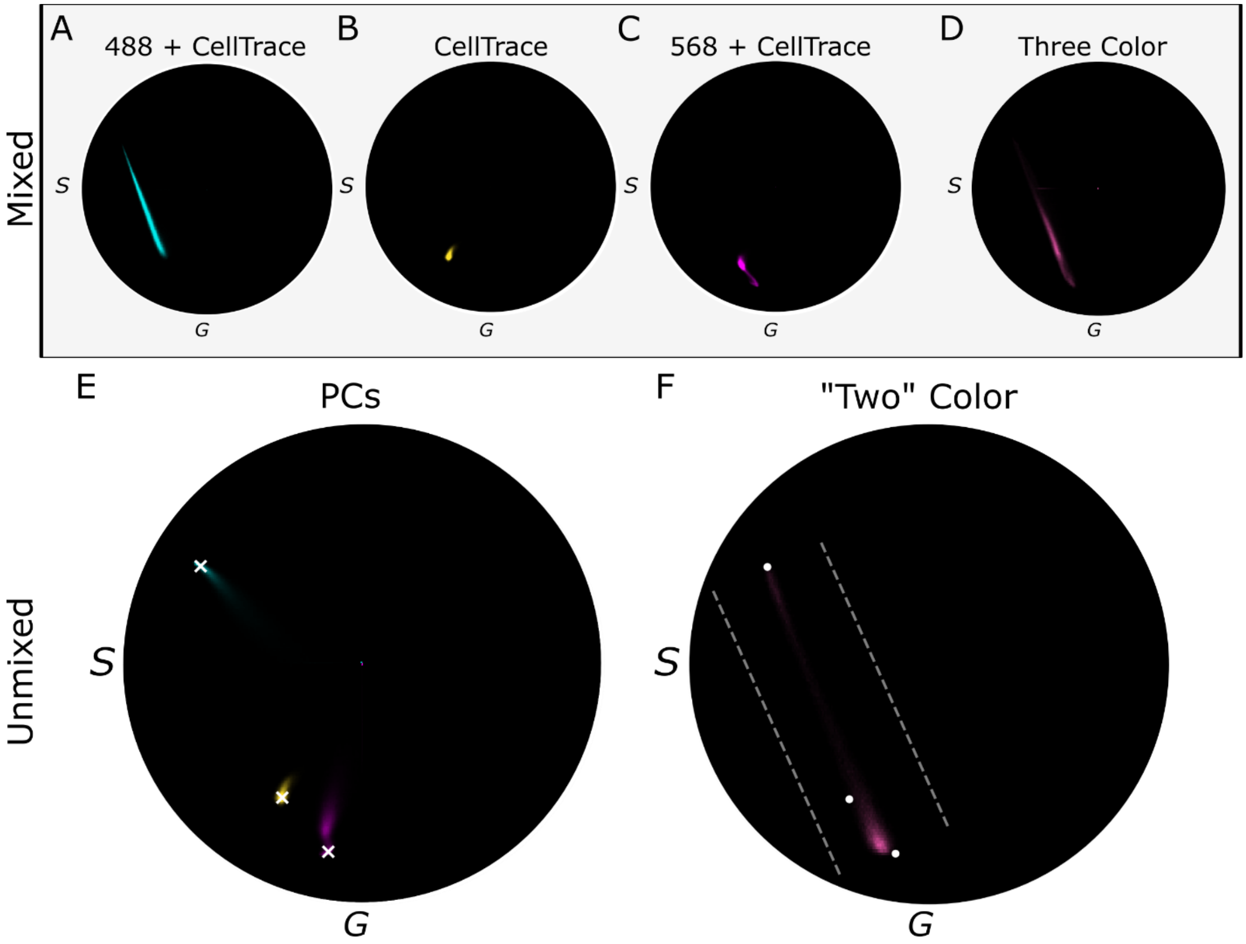

Fig. S7. Linear unmixing in phasor space A-C) Phasor clusters for the three-color experiment from Fig. 8. The presence of CellTrace results in pixels which contain contributions from both dyes. This leads to PC clusters (A,C) which are smeared between the phasor coordinates for pure AF488/568 and CellTrace itself (B). D) Similarly, the three-color sample exhibits a mixed phasor cluster which is spread out due to CellTrace.

E) Linearly un-mixed multispectral images lead to PC clusters free of contamination from CellTrace for AF488 (cyan) and AF568 (magenta). These unmixed images allow for accurate determination of the PC coordinates (white markers). F) Using the linear combination properties of phasors, we unmixed the three-color sample resulting in a mixing cluster which exhibits two clear populations near the 488 and 586 PC coordinates (top and bottom white dots), without influence of the CellTrace (middle white dot), indicating the expected minimal mixing.

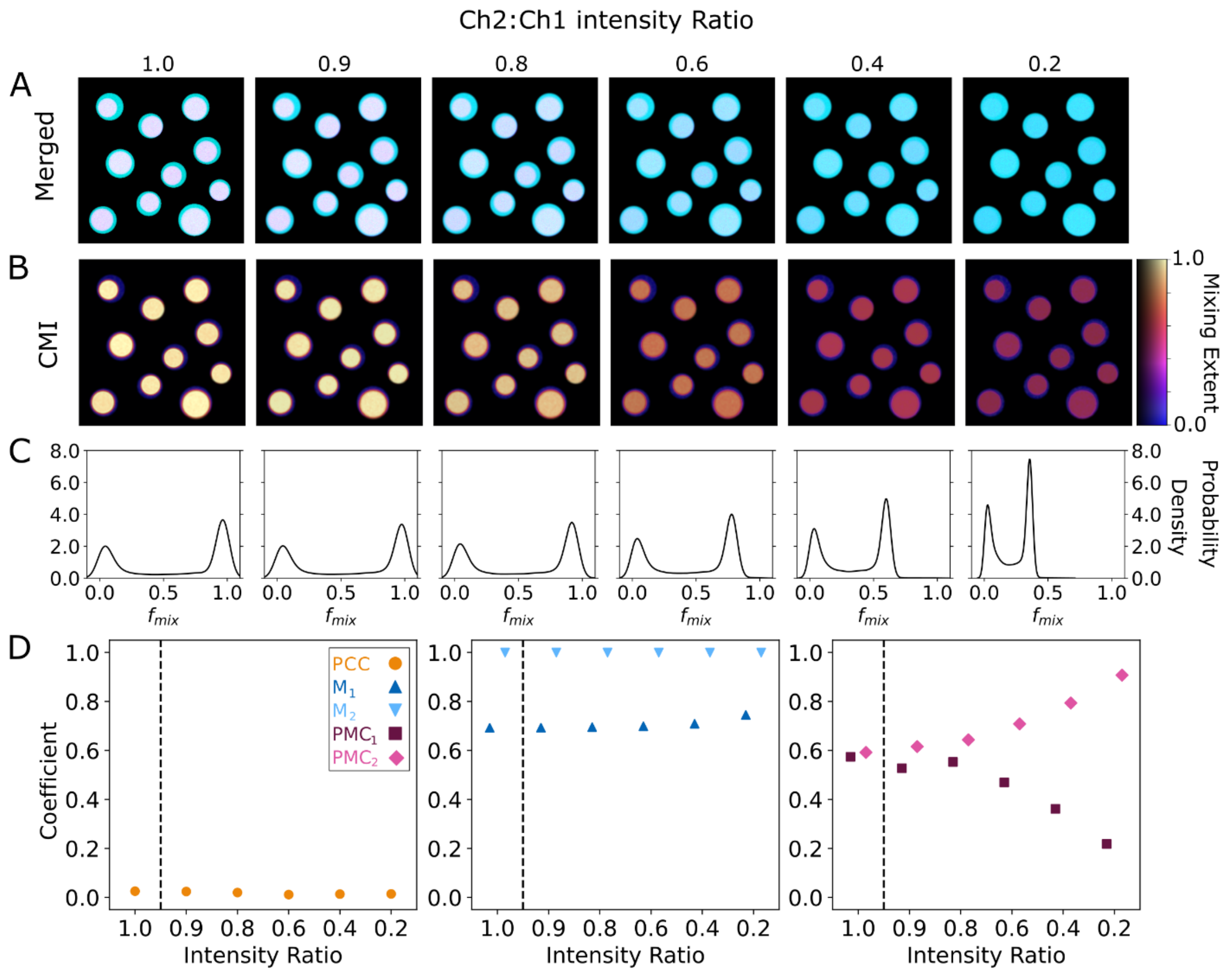

Fig. S8. Intensity Imbalance Effects on PMC The input images from Figure 1C were used to simulate an intensity imbalance between the two channels by decreasing the relative intensity of the magenta channel in Fiji. A) Pseudo-color merged images at decreasing ratios of Ch2:Ch1 intensity: from 1.0 (Figure 1C) to 0.2. B) Color Mixing Images for each intensity ratio from A). As the relative Ch2 intensity decreases from left to right, both the mixing and the summed intensity decrease, as indicated by the increasingly cooler color and lower brightness. To illustrate the decrease in intensity, the maximum brightness for each CMI was scaled to the maximum intensity of the control image (left). C) The mixing distribution for each intensity ratio. As the relative Ch2 intensity decreases, the distribution shifts to the left because of the imbalance of the signals. In particular, the peak in f_{mix} which is near 1.0 in the balanced scenario (left) shifts to ~0.4 in the most imbalanced scenario (right). This latter peak reflects pixels which contain a 20:80 mix of Ch2 and Ch1, rather than 50:50. D) PCC (left), $M_{1,2}$ (middle) and $PMC_{1,2}$ measurements for each intensity ratio. While PCC and $M_{1,2}$ do not vary much in this instance, $PMC_{1,2}$ exhibits fairly significant sensitivity to the signal imbalance: $PMC_{1,2}$ is most accurate when the signal levels of the two channels are within ~20% of each other.

Table S1. Image acquisition settings for data corresponding to Fig. 5A

	488 Power	561 Power	Offset	Gain (V)	Pixel Dwell Time (µs)	Pinhole (nm)	Line Averaging
Single-Color	0.8	0.1	0	680	8	112	4
Double-color	0.8	0.1	0	680	8	112	4

Table S2. Image acquisition settings for data corresponding to Fig. 5B and Fig. 6.

	488 Power	561 Power	Offset	Gain (V)	Pixel Dwell Time (µs)	Pinhole (nm)	Line Averaging
Single-Color	0.3	1.5	375	750	0.7	60	8
Double-color	0.3	1.5	375	750	0.7	60	8

Table S3. Image acquisition settings for data corresponding to Fig. 5C.

	488 Power	561 Power	Offset	Gain (V)	Pixel Dwell Time (µs)	Pinhole (nm)	Line Averaging
Single-Color	0.5	1.7	0	800	8	56	4
Double-Color	0.5	1.7	0	800	8	56	4

Journal of Cell Science • Supplementary information

Table S4. Image acquisition settings for data corresponding to Fig. 7. For all label combinations, images were acquired using laser powers in the following combinations: [(488, 561)] = [(0.01,0.1), (0.06,0.3), (0.12,0.6), (0.18,0.9), (0.24,1.2), (0.3,1.5)] %.

	Offset	Gain (V)	Pixel Dwell Time (μs)	Pinhole (nm)	Line Averaging
Single-Color	375	750	0.7	60	8
Double-color	375	750	0.7	60	8

Table S5. Image acquisition settings for data corresponding to Fig. 8.

Sample	488 Power	561 Power	Offset	Gain (V)	Pixel Dwell Time (μs)	Pinhole (nm)	Line Averaging
LAMP1	2.0	0.3	400	700	1.3	56	8
TOMM20	2.0	0.3	400	700	1.3	56	8
LAMP1+TOMM20	2.0	0.3	400	700	1.3	56	8
LAMP1+CellTrace	0.8	0.1	0	700	5	56	8
TOMM20+CellTrace	0.8	0.1	0	700	5	56	8
CellTrace	0.8	0.1	0	700	5	56	8
LAMP1 + TOMM20 + CellTrace	0.8	0.1	0	700	5	56	8

Journal of Cell Science • Supplementary information
